# Dual-step photo-induced self-assembled hydrogel for endogenous oral mucosal wound healing

**DOI:** 10.1038/s41377-025-01837-7

**Published:** 2025-05-15

**Authors:** Shaojun Fang, Qiangqiang Zhou, Mengqi Zhou, Changyi Li, Huaxing Xu, Hongyu Tang, Wanlu Zhang, Ruiqian Guo, Xiaoling Wei, Rongjun Zhang

**Affiliations:** 1 Department of Optical Science and Engineering, Key Laboratory of Micro and Nano Photonic Structures (MOE), Shanghai Engineering Research Center of Ultra-Precision Optical Manufacturing, School of Information Science and Technology, Fudan University, Shanghai, 200433 China; 2 Department of Endodontics, Shanghai Stomatological Hospital and School of Stomatology, Fudan University, Shanghai, 200001 China; 3 Shanghai Key Laboratory of Craniomaxillofacial Development and Diseases, Shanghai Stomatological Hospital and School of Stomatology, Fudan University, Shanghai, 200001 China; 4 Academy for Engineering and Technology, Fudan University, Shanghai, 200433 China; 5 Institute for Electric Light Sources, Fudan University, Shanghai, 200433 China

**Keywords:** Biomaterials, Polymers

## Abstract

By introducing piezoelectric materials into hydrogel oral dressings, a microelectric field could be generated under stress stimulation, thus facilitating oral wound healing. However, to adapt to the moist and dynamic environment of the oral cavity, traditional “step-by-step” synthesis often requires the combination of materials with different functionalities. Given the property differences between these materials, this strategy typically involves complex experimental procedures and unnecessary energy consumption. In this study, with the concept of “integrated construction”, we innovatively proposed a dual-step photo-induced method and successfully fabricated composite hydrogels with excellent performance. We introduced abundant oxygen vacancies into ZnO, leveraging the enhanced interface dynamics to achieve sustained photo-induced effect. With a double-network polymer framework as a template, this method could achieve the photo-induced spontaneous in-situ synthesis of polydopamine (PDA) within hydrogel without any extra special experimental conditions and complex operation procedures. We conducted a thorough analysis of the mechanism underlying this photo-induced method and applied the as-prepared hydrogel for the treatment of oral wounds, which significantly accelerated the healing process due to the outstanding comprehensive performance of hydrogel. These results suggest novel ideas and theoretical support for the facile construction of high-performance hydrogels based on photodynamic principles, demonstrating immense potential for future applications in wound dressings.

## Abstract (web summary)

A novel dual-step photo-induced self-assembly hydrogel based on defect engineering and interfacial effects has been developed for the endogenous treatment of oral mucosal wounds.

## Graphical abstract

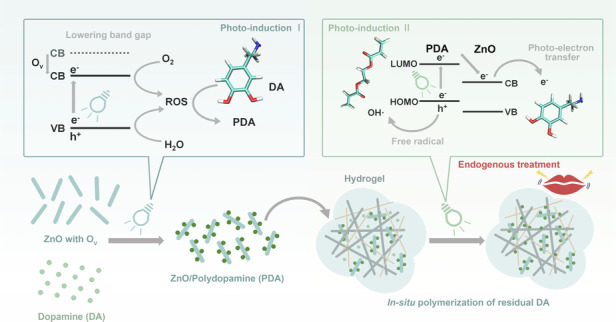

## Introduction

Oral wound healing is a crucial physiological process that involves the cellular inflammatory response, granulation tissue proliferation and regenerative tissue remodeling, and part of the repair process is triggered by bioelectric fields around the wound^1–3^. Traditional tissue engineering treatment includes three elements: cells, factors, and scaffolds, which require complex synthesis processes and may lead to side effects and drug resistance^4^. Hence, an increasing number of studies were searching for promoting wound healing methods by mimicking the internal repair patterns of biological systems. Recent studies have found that a weak electric field surrounds wounds as they form, which promotes cell proliferation and differentiation^1,5^. However, in the presence of factors such as inflammation, the microelectric field would be disrupted or shielded, thus slowing down the healing processes greatly^6,7^. Consequently, the composite dressings of nanogenerators are regarded as an endogenous therapy method to provide the biomimetic microelectric field and restore the disrupted wound environment, resulting in accelerating the oral wound healing process^8–10^.

As a typical nanogenerator material, ZnO has attracted generous attention for its unique photopiezoelectric and biocompatible properties^11–13^. It’s well known that ZnO has been approved by the U.S. Food and Drug Administration with good biocompatibility^14^. Based on the asymmetric arrangement of hexagonal wurtzite structure, ZnO could generate a net polarization and thus an electric field under mechanical stress, which could further produce reactive oxygen species (ROS) to kill pathogenic bacteria^15–17^. Hence, many studies capitalized on its piezoelectric properties for providing a biomimetic electric field around wounds, thereby facilitating endogenous wound healing^18–20^. Based on this, ZnO is regarded as one of the most suitable nanogenerator materials for endogenous oral mucosal wound healing. In contrast, though ZnO has exhibited remarkable optoelectronic and optical catalysis properties in various technological applications, little research in the oral wound healing field explores the photoapplication of ZnO^21–23^. The reason is that pure wurtzite ZnO displays a wide band gap of 3.4 eV results in a UV absorption band^24^, which is not only harmful to skin cells but also requires high energy for photo-activation. Thereby, the exploration of ZnO’s optical performance in hydrogel construction is still in its infancy, which remains a challenging aspect of current research.

Additionally, to fit the wet and dynamic environment of the oral cavity^25^, the endogenous therapy of oral wound always works through building composite hydrogel dressings, which are required to possess functions of excellent mucosa-fit, maintaining a moist environment, and indicating excellent biocompatibility^26,27^. To achieve these functional properties, composite hydrogel dressings always consist of several parts, including polymer skeletons, functional organic compounds, and inorganic nanoparticles. As one kind of inorganic materials, the poor contact between ZnO and polymer hydrogels further hinders the construction of suitable dressings for oral wound healing^28^. Hence, an organic coating at ZnO surface is regarded as an effective way to improve the organic-inorganic compatibility. As a versatile conversion coating, polydopamine (PDA) exhibits not only excellent adhesion ability, but also friendly biocompatibility and outstanding photoelectric and photothermal performance^29–31^, which could be utilized as a functional organic compound to effectively enhance the wet adhesion abilities of hydrogel dressings in the oral cavity. Meanwhile, PDA could form hydrogen bonds, electrostatic interactions, and *π*–*π* interactions with polymer frameworks with abundant functional groups like catechol, imine and amine, which further achieve the conjugation and immobilization of nanoparticles in polymer skeletons^5^.

However, PDA exhibits low solubility in both polar and non-polar solve in conventional conditions, and traditional preparation of PDA-coating strictly requires controlling the pH, temperature and UV irradiation catalysis^32–34^, which greatly influence its high-effective synthesis. Furthermore, due to the structural damage that UV and alkaline environments may cause to hydrogels, traditional methods often involve constructing PDA-coated materials at first and then incorporating them into a polymer framework to form composite hydrogels. This ex-situ “step by step” method typically requires operations such as centrifuging and drying, making the experimental process lengthy and the operations cumbersome, which also results in significant energy consumption. Moreover, many potentially toxic and recalcitrant chemicals used in the synthesis, such as initiators, enzymes, or catalysts, render the resultant hydrogels less biocompatible. Therefore, it is essential to develop an efficient, convenient, and energy-saving composite construction method. Recently, photoelectron transfer (PET) has been attempted to polymerize dopamine (DA) into PDA^32^, but the mechanism and practical application of PDA spontaneous formation and synergetic effect at coating interfaces are still mysteries.

Herein, we skillfully fabricated a novel dual-step photo-induced method to build the in-situ self-photopolymerized PVA/PEGDA/PDA/(ZnO-D/PDA) (PPPZ) hydrogel dressings for endogenous oral wound healing. Using a double-network polymer framework as a template, we successfully achieved in-situ assembly of PDA within the hydrogel through a continuous synthesis process involving a dual-step photo-induced effect. The well-designed method achieves the “integrated construction” of hydrogels within the same system and results in the uniform distribution of PDA and ZnO-D in PPPZ, which brings hydrogel excellent mechanical properties. The PPPZ displayed excellent biocompatibility and antibacterial properties, which could greatly promote the proliferation and differentation of *Human Oral Fibroblasts* (*HOFs*). In the rat model of full-thickness buccal mucosa defects, PPPZ exhibited better therapeutic effects than commercial dressings. Through experimental and theoretical ways, we further elucidated the mechanism of the dual-step photo-induced method and the intrinsic process of PPPZ in wound healing. This study presents a fresh design strategy and theoretical support for effective synthesized oral wound dressings based on the comprehensive properties of each composite, and the as-prepared hydrogels exhibit the potential to fulfill clinical demands for oral wound dressings and address various issues in wound treatment.

## Results

### Dual-step photo-induced hydrogel synthesis and characterization

**Fig. 1:**
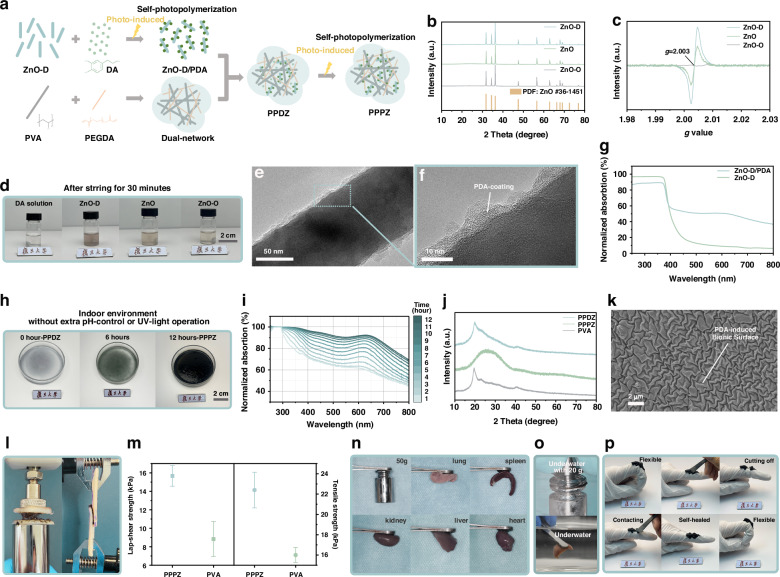
**The materials characterization of PPPZ.** **a** Schematic representation for the preparation of PPPZ. **b** XRD and **c** EPR spectrum of ZnO-D, ZnO, and ZnO-O. **d** The photos of DA solution color changing after mixing with ZnO-D, ZnO, and ZnO-O. **e**, **f** TEM images of ZnO-D/PDA. **g** UV-visible spectrum of ZnO-D and ZnO-D/PDA. **h** The photos of hydrogels color changing. **i** The UV-visible curves of hydrogels at different time (1 h–12 h). **j** XRD spectrum of PPDZ, PPPZ and PVA. **k** SEM image of PPPZ surface. **l**, **m** Lap-shear and tensile strength tests of PPPZ. **n**, **o** Adhesion abilities of PPPZ in air and water. **p** Flexibility and self-healed ability of PPPZ

With the advancement of technology in the manufacturing industry, people have gradually replaced the integration of complex and cumbersome micro-components with “integrated construction”, thereby achieving efficient and holistic production manufacturing. Similarly, we have achieved a simple “integrated construction” of hydrogels using a dual-step photo-induced method. The synthesis processes of photo-induced hydrogels are shown in Fig. 1a. First, we successfully fabricated ZnO materials with rich oxygen vacancies (O_V_), which were named ZnO-Defect (ZnO-D). Meanwhile, the PVA and PEGDA were designed as a dual-network framework, which could not only provide mechanical strength and flexibility but also serve as a template for the assembly of PDA within the network space. After the initial photo-induced step, the hydrogel matrix rich in DA, PVA, and PEGDA was directly mixed with ZnO-D/PDA solution, forming hydrogel PVA/PEGDA/DA/(ZnO-D/PDA) (PPDZ). The PPDZ undergoes continuous internal reactions under the influence of the second photo-induced step, resulting in in-situ PDA formation, thus turning to the final composite hydrogel PVA/PEGDA/PDA/(ZnO-D/PDA) (PPPZ). All processes only require simple stirring or heating, without the need for multi-step separate synthesis or additional operations, which can greatly simplify the preparation process of hydrogels. Generally, the dual-step photo-induced method worked on two parts: ZnO-D/PDA formation and PPPZ transition.

To explore the influence of O_V_ in the photo-induced process, we synthesized untreated ZnO (ZnO) and ZnO with low O_V_ concentration (ZnO-Oxide, abbreviated to ZnO-O) as control groups^35^. As shown in Fig. 1b, the X-Ray Diffraction (XRD) peaks of ZnO-D, ZnO, and ZnO-O are in good agreement with the normative diffraction patterns of ZnO (JCPDS: #36-1451), and no further secondary phases are found, which indicates the successful synthesis of three samples. Meanwhile, compared to the ZnO patterns, it could be observed in Fig. S1 that the diffraction peaks of ZnO-D shift to the high-angel direction while those of ZnO-O move to the low-angel direction, corresponding to the residual stress by the changes of O_V_^36^. The structure was studied further by Raman spectra (Fig. S2); the uplift spectrum curves of ZnO-D indicate the strong fluorescence interference, which was caused by the abundant vacancies at surface^37^. As shown in Fig. S3, the strong peak at around 436 cm^−1^ is related to the characteristic Wurtzite E_2_(high) mode of ZnO, further verifying the crystal structure of samples^38^. Meanwhile, the E_1_(LO) peak located at 572 cm^−1^ is always assigned to O_V_, while the intensity of ZnO-D displays the highest values due to its highest defect concentration. Furthermore, the Electron Paramagnetic Resonance (EPR) results in Fig. 1c show that ZnO-D crystals possess much more O_V_ due to its strongest O_V_ characteristic peaks at a *g* value of 2.003 among three samples^39^. In addition, the Scanning Electron Microscopy (SEM) morphologies of samples all exhibit the classical shape of nanorods (Fig. S6), while the High-Resolution Transmission Electron Microscopy (HRTEM) image reveals the lattice spacing of 0.257 nm, corresponding to the (002) plane of ZnO (Fig. S7). Overall, the characteristic analyses above illustrate the successful synthesis of ZnO-D, ZnO and ZnO-O.

As mentioned above, we respectively dissolved ZnO-D, ZnO and ZnO-O in DA solution by stirring for 30 mins without any extra operation. To clearly observe the differences in samples, the concentration of ZnO-D, ZnO and ZnO-O was set as 0.02% (w/v). The results in Fig. 1d indicate that the color of the samples turned from transparent to red, which is the mark that DA polymerized to form PDA. Notably, it could be clearly observed that the ZnO-D/DA solution displays the darkest colour, which means the highest efficiency of ZnO-D in facilitating the polymerization of DA. Meanwhile, the Fourier Transform Infrared Spectroscopy (FTIR) results (Fig. S8) illustrate that the crystal structure of ZnO-D was still maintained, while the FTIR spectrum of ZnO-D/PDA shows sharper peaks at ~500 cm^−^^1^ and small uplift curves at ~1500 cm^−^^1^, which correspond to the PDA-coating^40,41^. In the TEM images (Fig. 1e, f), the PDA-coating presents an amorphous phase at ZnO-D surface, further verifying the successful ZnO-D/PDA formation after initial photo-induced process. In contrast, the surface of the ZnO-O/PDA material shows no significant coating layer, indicating that O_V_ would induce the formation of PDA (Fig. S27). In addition, the absorption of ZnO-D declines rapidly beyond the UV-light range, while the ZnO-D/PDA displays higher absorptivity in the visible wavelength range (Fig. 1g), which could be attributed to the excellent photoelectricity effect of PDA^33,42^. Overall, the more O_V_ bring stronger catalytic ability to ZnO-D, resulting in more effective ZnO-D/PDA formation and improved visible-light absorption.

During the second photo-induced process, the as-prepared PPDZ hydrogel was placed under natural environmental conditions. After 12 hours, the color of PPDZ turned from light blue into dark black gradually (Fig. 1h), which means the unpolymerized DA could in-situ form PDA^30^. The UV-Visible absorption-time curves in Fig. 1i verifies the enhanced visible-light absorption over time, which leads to the color change and exhibits potentials of long-wavelength response in biomedicine fields. Furthermore, the XRD spectrum of PPDZ has a clear characterized peak of PVA, while the slight protrusion of curves at ~23° could be contributed to the partial-polymerized PDA (Fig. 1j). The curves of PPPZ show the broad peak centered at the same position, corresponding to a spacing of ~3.8 Å which belongs to the typical π-π stacked phenyl unit, indicating the in-situ formation of PDA in hydrogel^43^. Moreover, in the FTIR spectrum (Fig. S9), the features of PPPZ between 3000 and 3400 cm^−1^ correspond to the aromatic O-H stretching vibration region as well as the amine functionality patterns at 1593 cm^−1^ and 1498 cm^−1^ respectively, which could further verify the formation of PDA under the stimulation of lights. Notably, the peak located at 1650 cm^−1^ belongs to the C=C bond of PEGDA, which could hardly be observed in the spectra of PPPZ, illustrating that further polymerization of PEGDA also happened. In a word, the second photo-induced process achieves the effective in-situ synthesis of PDA in the hydrogel frameworks.

In addition, the SEM images in Fig. S10 show the uniform and smooth surface of PPPZ, which means the excellent compatibility between ZnO-D/PDA and dual-network hydrogel. In a larger scale (Fig. 1k), the surface of PPPZ displays ridge-like morphology, which was caused by the stress release during the polymerization of DA^34^, demonstrating the templating effect of the dual-network in guiding the uniform assembly of PDA. This bionic structure could greatly increase the contact area between dressings and oral wounds, resulting in outstanding adhesion. The EDS mapping results (Fig. S12) further verify the uniform distribution of each element in PPPZ, indicating the successful synthesis through the dual-step photo-induced reaction.

As mentioned above, stable and robust adhesion under wet conditions is crucial for the application of hydrogels in oral wounds. The in-situ formed PPPZ displays distinctive performance as dressings for oral wounds. We compared the contact angel between PPPZ and pure PVA, as shown in Fig. S13, PPPZ displays an improved value of 35.1°, while which of PVA is only 50.5°, illustrating the enhanced wet adhesion ability^44,45^. Moreover, we determined the adhesion strength of PPPZ by measuring the force required for the hydrogel to depart from the substrate surface. As depicted in Fig. 1l, m, the PPPZ exhibits substantial enhancements in both shear and tensile stress compared to PVA, which can be attributed to its internal double-network framework and the diverse covalent bond bindings with PDA.

Because of the excellent adhesion abilities of PDA, the PPPZ exhibits satisfactory adhesive properties to different materials and tissues such as 50 g weight, lung, spleen kidney, liver, and heart (Fig. 1n). The outstanding performance maintained even in the under-water environment, in which PPPZ still performed stable adhering to 20 g weight and liver tissues (Fig. 1o). In addition, the PPPZ adhered tightly to the gloved finger without any detachment even after stretching; after cutting and simply physical connecting, it exhibits rapid self-healed abilities cand still keep flexible (Fig. 1p and Supplemental Video 1). During in vivo testing (Fig. S16 and Supplemental Video 2), the PPPZ hydrogel also demonstrated excellent adhesion properties, maintaining stability even under the flushing of artificial saliva. Furthermore, we conducted tests on the wet adhesion capacity of the same PPPZ hydrogel at different temperatures (4 °C, 25 °C, and 37 °C) to assess its ability to adapt to the potential dynamic temperature changes that may occur in the oral cavity. The experimental results (Fig. S17 and Supplemental Video 3) indicate that PPPZ can effectively adhere to the skin surface, demonstrating its excellent dynamic adhesion performance. Additionally, the results of tensile testing also show that the hydrogel exhibits outstanding strain stability, meeting the practical requirements for use in the oral cavity (Figs. S19 and S20). In a word, the dual-step photo-induced method effectively achieves the uniform distribution of both PDA and ZnO-D in the hydrogel, which brings PPPZ excellent mechanical performance for the complex environment of the oral cavity, thus showing immense potential as wound dressings.

### Dual-step photo-induced mechanism

**Fig. 2:**
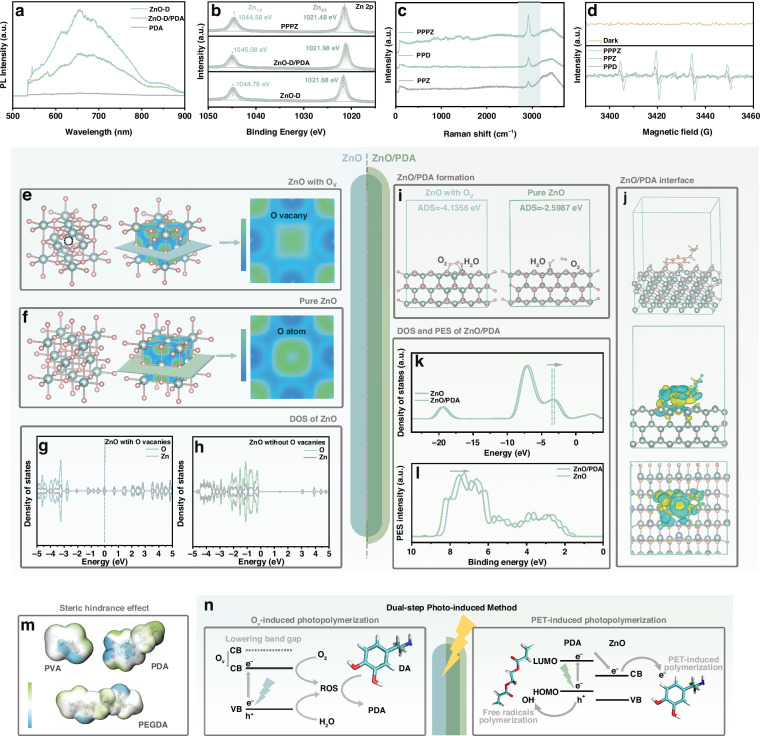
**The mechanism of dual-step photo-induced method.** **a** PL spectra of ZnO-D, PDA and ZnO-D/PDA. **b** Zn 2p spectra of PPPZ, ZnO-D/PDA and ZnO-D. **c** Raman results of PPPZ, PPZ, and PPD. **d** EPR spectra of PPPZ, PPZ, and PPD. ELF results of (**e**) ZnO with O_V_ and (**f**) pure ZnO. DOS spectra of (**g**) ZnO with O_V_ and (**h**) pure ZnO. **i** O_2_/H_2_O adsorption energy of ZnO with O_V_ and pure ZnO. **j** Deformation charge density of ZnO-O_V_/PDA. **k** DOS and **l** PES spectra of ZnO-O_V_ /PDA and ZnO-O_V_. **m** Electrostatic potential of PVA, PEGDA, and DA. **n** Mechanism schema of dual-step photo-induced process

The analysis above has proved the excellent performance of PPPZ constructed by the simple dual-step photo-induced method, which leads to an interesting question: how does this highly easy-made and effective photo-induced method work? According to recent studies, PET was the key to actualize the polymerization of DA^32^, so we explore the electron transfer ability of ZnO-D/PDA by experimental methods. The Photoluminescence (PL) spectra of ZnO-D, ZnO-D/PDA and PDA were displayed in Fig. 2a, it could be clearly observed that ZnO-D has a broad high-strength band due to its abundant O_V_, while the spectrum of PDA reveals almost no PL intensity because of its high degree of polymerization and complex stacking structures^46^. After initial photo-induced binding, the ZnO-D/PDA display a quenching of fluorescence, which means the rate of electron-hole recombination in ZnO-D/PDA is effectively suppressed due to the enhanced photoelectron tansfer^47^.

In addition, the X-ray Photoelectron Spectroscopy (XPS) tests were hired to further uncover the formation mechanism of the photo-induced method. The variation of the binding energy corresponding to a certain element illustrated the charge migration across the interface. If the binding energy displays a positive-shift illustrated the charge exportation, otherwise illustrated charge importation^48^. In order to further make sure the PET effect of ZnO-D/PDA works in hydrogel, we set two other types of hydrogel as control groups: PVA/PEGDA/ZnO-D (PPZ) with only ZnO-D and PVA/PEGDA/DA (PPD) with only DA. As shown in Fig. 2b, the characteristic peaks of Zn 2p in ZnO-D/PDA exhibit a positive shift compared to ZnO-D, which could be attributed to the electron transfer after ZnO/PDA interface binding while PDA occupied the position of O_V_ and attracted the electrons of Zn atom. Furthermore, the spectra of PPZ (Fig. S14) further illustrate the negligible influence of PVA/PEGDA framework in ZnO-D valances since the Zn 2p peaks display no shift compared to those in ZnO-D. Notably, compared to ZnO-D/PDA and ZnO-D, the Zn 2p spectra of PPPZ exhibit a negative shift, indicating the inflow of electrons in ZnO-D after photo-induced binding^48^. In contrast, the N 1 s peaks of PPPZ split to two main peaks and shifted to higher values than PPD (Fig. S15), corresponding to the outflow of electrons in PDA^49^. The peaks shift indicates the continuous photoelectrons migration from PDA to ZnO-D thus forming PET-induced PDA in hydrogel framework under light irradiation in PPPZ formation, which could explain the gradually enhanced long-wavelength absorption.

Meanwhile, compared to those of PPZ and PPD, the Raman results in Fig. 2c illustrate the highest intensity of PPPZ, which could correspond to the surface enhancement Raman spectrum (SERS), which mainly depends on the exciton transfer and resonance at the surface to amplify the Raman signals^33^, confirming the quick carriers transfer in ZnO-D/PDA interface. Thus, the enhanced PET effects in both composite ZnO-D/PDA and PPPZ were verified as one of the reasons for how photo-induced method works. Moreover, the EPR spectra in Fig. 2d display that the shapes of PPPZ curves were accorded with those of typical hydroxyl free radicals curves after 10 minutes illumination, with nearly no peak shown in spectrums of PPD and PPZ. The results could be explained by the free radical generation by PDA under light illumination^50^, facilitating the further cross-linking of PEGDA.

Back to when the initial photo-induced method worked on ZnO-D to build ZnO-D/PDA, the Density Functional Theory (DFT) calculations were constructed to analyze the underlying causes^51^. As shown in Fig. 2e, f, the Electron Localization Function (ELF) results of pure ZnO display high electron distribution density around O atom due to its strong electronegativity. After introducing O_V_ defects, there is a strong electron delocalization area around the vacancy, facilitating the photoelectron transfer and resulting in high carrier mobility^52^. In addition, the Density of States (DOS) spectra of pure ZnO in Fig. 2h indicate a clear band gap between conductive band (CB) and valence band (VB), resulting in difficult photoexcition transition and fast carrier recombination. In contrast, the Fermi energy level lifts up with O_V_ introduction, while the defect state density significantly increases around Fermi level energy due to the relaxing electron-withdrawing effect of O_V_ and electron doping (Fig. 2g). The electronic local areas near the Fermi level provide extra pathways for photoelectron transfer, thus achieving more effective carrier separation.

Furthermore, in the first photo-induced step, the ZnO with O_V_ displays a lower O_2_/H_2_O absorption energy of −4.1356 eV while which of pure ZnO is −2.5987 eV (Fig. 2i). The synergism of fast photoelectron transfer and improved O_2_/H_2_O absorption results in high-efficient formation of photo-generated ROS, thus catalyzing the polymerization of DA to form ZnO/PDA. At the same time, the lower adsorption energy between O_V_ and DA monomers also allows the active sites to remain continuously exposed, maintaining the dynamic equilibrium of the reaction and enabling the entire process to proceed (Fig. S28). Additionally, the deformation charge density in Fig. 2j further reveals the charge transfer between ZnO and PDA at ZnO/PDA interface. As displayed, due to the existence of O_V_ with electron delocalization effect, the binding of PDA and O_V_ formed the increasing charge density in the bonding area and resulted in the electron transfer from Zn atom to PDA, verifying the previous results of XPS peaks shift. The DOS in Fig. 2k further illustrate the uplift energy level of ZnO/PDA compared to ZnO, which corresponds to the improved electron transfer, indicating the enhanced carrier separation of ZnO/PDA interface. Moreover, the Photoelectron Spectrum (PES) peaks of ZnO/PDA (Fig. 2l) show a negative shift, demonstrating the easier photoelectron excitation after binding, which causes highly effective photoelectron excitation and separation with the combined action of DOS results. In addition, the electrostatic potentials of PVA and PEGDA macromolecules both display strong polar groups, resulting in effective steric hindrance effects for finite PDA formation at ZnO surface (Fig. 2m). These effects effectively restrict the agglomeration of PDA within the hydrogel network and guides the in-situ assembly within the network skeleton template, resulting in the formation of a uniform biomimetic morphology at the surface. The analysis above was consistent with how XPS spectrum displayed, illustrating the mechanism of quick formation and electron transfer in ZnO/PDA.

Overall, the mechanism schema of dual-step photo-induced polymerization is shown in Fig. 2n, which could be divided into two parts: Ov-induced photopolymerization and PET-induced photopolymerization. In the initial photo-induced step, the abundant O_V_ in ZnO play three roles: enhancing interfacial active electron transitions, adsorbing H_2_O/O_2_ to participate in the reaction and continuously exposing reactive sites, resulting in ROS formation and DA polymerization. During the second photo-induced process, the ZnO/PDA interface indicates effective visible-light absorption and photoelectron separation, which results in generous active photoelectrons and free-radicals, thus facilitating the continuous in-situ photopolymerization. Additionally, the dual-network framework acts as a template for uniform in-situ polymerization of DA. The well-designed photo-induced method achieved even distribution of in-situ self-assembled PDA in hydrogel frameworks through a simple synthesis method, and the as-prepared hydrogels are in accord with the wet adhesion and self-healing demands of oral wound dressings.

### Antibacterial capacity and biocompatibility in vitro and in vivo

By modifying the optical properties of ZnO, we successfully utilized the dual-step photo-induced method and fabricated the PPPZ hydrogel dressings with excellent wet adhesion and self-healing properties. As mentioned, ZnO is one of the special materials with both photoelectricity and piezoelectricity. Hence, muscle activities in the oral cavity, such as chewing, can apply pressure to PPPZ, thereby stimulating the piezoelectric properties of the internal ZnO and forming an endogenous electric field around the wound. Based on this, we further explore the application of PPPZ in implementing endogenous therapy on oral mucosal wounds.

**Fig. 3:**
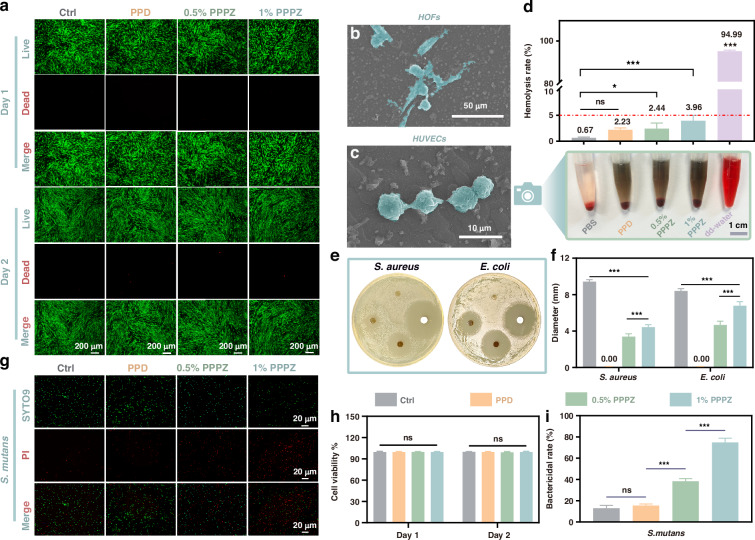
**Biocompatibility and antimicrobial properties of the piezoelectric hydrogel in vitro.** **a**, **h** Live/dead staining of *HOFs* after incubation in piezoelectric hydrogel extracts for 1, 2 d. *n* = 4. Scale bar: 200 μm. SEM photographs of *HOFs* (**b**) and *HUVECs* (**c**) adhering to the surface of 1% PPPZ hydrogel. **d** Evaluation of the hemoslytic properties of piezoelectric hydrogel in vitro. *n* = 4. **e** Photographs of antibacterial zones of *S. aureus* and *E. coli* around the samples. **f** Statistical diagram of antibacterial area. *n* = 3. **g** The live and dead bacteria were stained by SYTO 9 (green) and PI (red), respectively. Scale bar: 20 μm. **i** The antibacterial ratio of each group. *n* = 4

Firstly, excellent biocompatibility is an essential criterion for the ability of hydrogels to serve as effective wound dressing. Hence, the cytocompatibility of PPPZ was investigated with Human Oral Fibroblasts (*HOFs*). The staining images of living and dead cells (Fig. 3a, h) showed that the numbers of live cells cultured with PPD and different ZnO concentration (0.5% and 1%, respectively represents the value of m_ZnO_:m_PVA_) PPPZ gels all increases after both 1 day and 2 days, while nearly no dead cell was observed. In addition, good cell adhesion and surface hardness of hydrogels play key roles in hydrogel-tissue adhesion, which could be beneficial for cell expansion and function utilization. As displayed in Fig. 3b, c, the *HOFs* and *Human Umbilical Vein Endothelial Cells* (*HUVECs*) could grow well, and cells were connected by their pseudopod, respectively. Furthermore, the hemolytic analysis results of PPD (2.23%), 0.5% PPPZ (2.44%) and 1% PPPZ (3.96%) all indicate good blood compatibility while the values of their hemolysis rate were below 5% (Fig. 3d), which is the standard of blood compatibility enacted by ASTM: F756-00. Previous analyses strongly prove the outstanding biocompatibility of PPPZ hydrogel.

The in vivo safety assessment results demonstrated that the rats remained healthy during the subcutaneous implantation in the dorsal region. Visual observation revealed no obvious redness, swelling, or rupture in the wounded area, indicating good healing (Fig. S21). Over time, the material gradually degraded in vivo, with a gradual reduction in volume. Histological examination with Hematoxylin and Eosin (H&E) staining showed mild inflammatory cell infiltration at the implantation site of the 1% PPPZ hydrogel on day 4. However, no inflammatory cell infiltration was observed around the material on days 7, 14, and 28, and the hydrogel was tightly encapsulated by connective tissue (Fig. S22). These results indicate that the material exhibits excellent biosafety and favorable degradation properties.

Bacterial infection is a great threat to the oral wound healing, in order to explore the antibacterial performance of PPPZ, *Staphylococcus aureus* (*S. aureus*) and *Escherichia coli* (*E. coli*) were used as representative pathogen. The bacterial area was determined by coculturing bacteria with the PPD, 0.5% PPPZ and 1% PPPZ, while the antibiotic susceptibility tablet was set as a control group. The results of the inhibition zone (Fig. 3e) indicate that the antibacterial effect of PPD was not considerable. However, a considerable inhibition zone existed in the 0.5% PPPZ and 1% PPPZ groups. Significantly, the inhibitory zone diameters of 1% PPPZ against both bacteria were statistically larger than those of 0.5% PPPZ (*p* < 0.05), illustrating the antibacterial activity of piezoelectric ZnO-loaded hydrogels (Fig. 3f).

Considering the different bacterial environments in the oral cavity, the antibacterial activity against *Streptococcus mutans* (*S. mutans*) was further evaluated by Live/Dead staining. As shown in Fig. 3g, i, the fluorescent images were basically consistent with the previous results, while the 0.5% PPPZ (38.32%) and 1% PPPZ (74.81%) both display the enhanced antibacterial activity (PPD group is 15.39% and control group is 12.86%) which is in proportion to the ZnO concentration (*p* < 0.05 in 1% PPPZ). Previous research has proved that the excellent antibacterial activity of ZnO originates from the photo-pizeoelectric ability, while the ROS could be generated by the microelectric fields made by photoelectric- and piezoelectric-effect. The ROS could directly destroy the bacterial cell walls and internal structures, leading to leakage of cellular contents, while the microelectric fields would interfere with charge distribution of bacterial cell surface and physiological activities, thus leading to bacterial programmed cell death. Furthermore, our results in the in vitro cellular environment demonstrate that the ROS generated by 1% PPPZ under ultrasound irradiation does not significantly affect cell viability (Fig. S23). Overall, 1% PPPZ could effectively eliminate bacteria in the oral cavity and prevent further infection.

### Modulation of *HOFs* and *HUVECs* biological functions in vitro

**Fig. 4:**
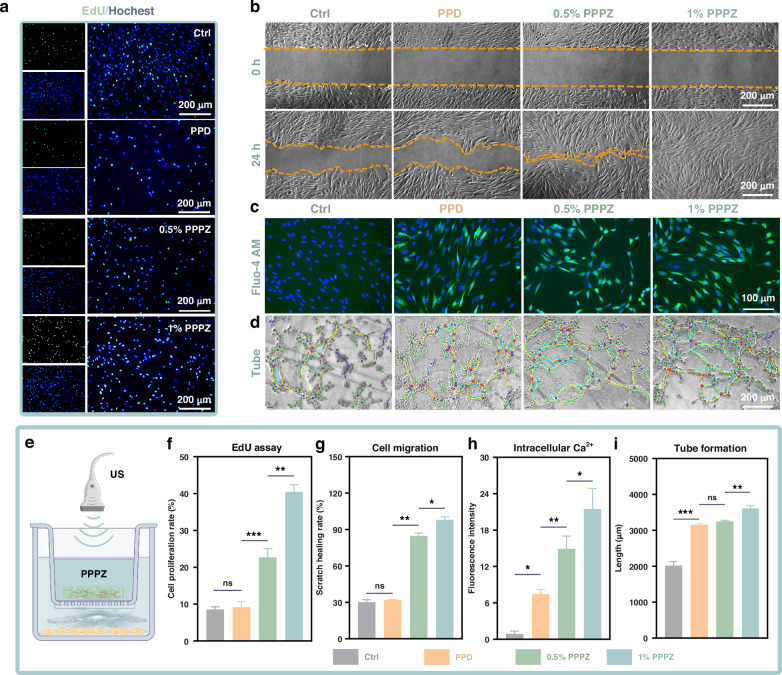
**Regulation of biological functions in*HOFs*and*HUVECs*by piezoelectric hydrogels.** **a** EdU staining to detect the effect of piezoelectric hydrogels on the proliferation of *HOFs*. Images show EdU-stained *HOFs* indicating cell proliferation (green), with nuclei labeled by Hoechst (blue). *n* = 4. Scale bars: 200 μm. Statistical analysis presents cell proliferation proportion of EdU for *HOFs* (**f**). **b** Microscopy images of cell migration scratch assay. Statistical analysis of the scratch test results displays the percentage of the wound closure area (**g**). Scale bars: 200 μm. **c** Intracellular calcium levels were measured using the fluorescent calcium indicator, Fluo-4 AM. Scale bars: 100 μm. Quantification of fluorescence intensity from Fluo-4 AM labeled calcium ions is shown (**h**). **d** Representative results of in vitro tube formation experiments on *HUVECs* by piezoelectric hydrogels. Scale bars: 200 μm. **i** Quantitative analysis of tube formation. **e** Schematic representation of piezoelectric hydrogels stimulating cells under ultrasound action. *n* = 3, *p < 0.05, **p < 0.01, and ***p < 0.001 indicated statistical difference and ns denoted no significant variation

As mentioned, ZnO could exhibit typical piezoelectric performance for facilitating the wound healing processes, in which the *HOFs* transfer and proliferation would happen around the wound to produce collagen and extracellular matrix components, resulting in enhanced wound mechanical strength and newborn tissue framework. In addition, the newborn vessels caused by vascular endothelial cell differentiation could provide enough O_2_ and nutrition for healing. Based on this, the promoting proliferation effect of PPPZ was tested by EdU proliferation technology and cell scratch technology under US stimulation. The EdU proliferation results in Fig. 4a show the significantly increasing proportion of EdU-positive cells (green fluorescence) cultured with 0.5% PPPZ and 1% PPPZ with the limited EdU-positive proportion in Ctrl and PPD groups, indicating the promotional DNA synthesis and proliferation of cells. In addition, the cell scratch results display the microscopic images of cell migration from 0 h to 24 h. As indicated in Fig. 4b, all hydrogel-based substances caused a positive effect on the scratch healing compared to the controls, while 1% PPPZ exhibits the best influence. In summary, PPPZ could play an important role in remarkably improving the transfer and proliferation of *HOFs*, which is of great significance for oral wound healing.

To further explore the impact of PPPZ on cell vital movement, we tested the Ca^2+^ levels of *HOFs* cultured with different hydrogels by Fluo-4 AM fluorescence probe. Compared to the controls, the Ca^2+^ levels of *HOFs* were significantly increased in 0.5% PPPZ and 1% PPPZ (*p* < 0.05), in which 1% PPPZ exhibits the best ability for Ca^2+^ levels regulation (Fig. 4c). This is consistent with the flow cytometry results (Fig. S24). To further investigate the intrinsic mechanism by which the hydrogel promotes phenotypic changes in cells, we used qPCR to detect the relative expression levels of key genes in the PI3K/AKT signalling pathway. The results show that compared to other groups, 1% PPPZ significantly upregulated the mRNA expression of PI3K, AKT, and mTOR (Fig. S25). Furthermore, the tube formation assay was conducted to confirm the angiogenesis properties of the different hydrogels. As shown in Fig. 4d, *HUVECs* cultured on 0.5% PPPZ and 1% PPPZ formed more endothelial meshes, tube structures and junctions compared to those in PPD and control group. The above results indicate the potential promoting effect of PPPZ on the activation and regulation of cellular functions, providing important cellular-level evidence for the application of piezoelectric materials in the field of biomedicine.

### Promotion of oral mucosal wound healing in vivo

**Fig. 5:**
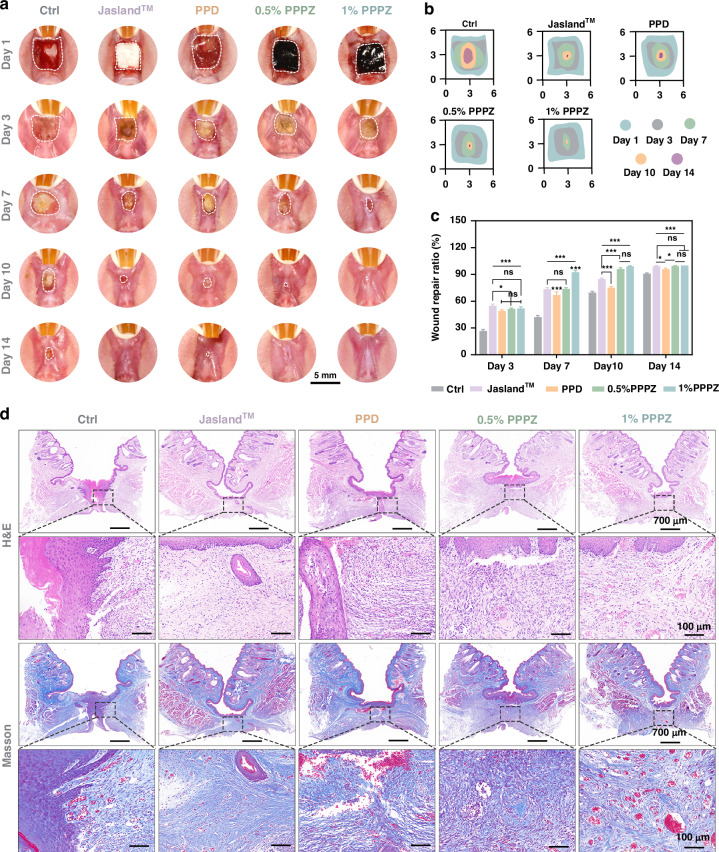
**Valuation of piezoelectric hydrogel on buccal mucosal wound healing in vivo.** **a** Representative photographs of wound healing in the control, Jasland^TM^, PPD, 0.5% PPPZ, and 1% PPPZ groups on days 1, 3, 7, 10, and 14 after treatment. **b** Depiction of wound healing area and (**c**) quantitative analysis of wound healing rate in different treatment groups, *n* = 4. **d** Representative H&E and Masson staining images of oral mucosal wounds in the control, Jasland^TM^, PPD, 0.5% PPPZ, and 1% PPPZ groups on day 14. *p < 0.05, **p < 0.01, and ***p < 0.001 indicated statistical difference and ns denoted no significant variation

Based on previous analysis, the PPPZ hydrogels display outstanding mechanical and in vitro biological properties. In order to access the efficacy of PPPZ in oral mucosal wound healing in vivo, we utilized the rat model of full-thickness buccal mucosa defects. As mentioned before, we conducted in vivo adhesion-related tests, and found that the PPPZ could firmly adhere to the wound site after being placed for 24 hours and even under artificial saliva rinsing (Fig. S16 and Supplemental Video 2), indicating that the PPPZ can effectively be applied to actual wound healing. Figure 5a shows photographs of the wounds at distinct time intervals, verifying a significant enhancement in the progression of oral wound healing in the 1% PPPZ group compared with any other groups. Notably, all treatment groups displayed enhanced properties than control group (*p* < 0.05), the rates of wound healing in the Jasland^TM^ and 1% PPPZ groups were both higher than others with similar values at Day 3 (Fig. 5b, c). On Day 7, 1% PPPZ group gained superiority, and this difference was most notable after this time point. The mucosa has fully healed without scars in 1% PPPZ group at Day 10, while the unhealed wounds still existed in control, Jasland^TM^, PPD and 0.5% PPPZ groups.

Furthermore, histological analysis of wound tissues on Day 14 was conducted to comprehensively evaluate the impact of different dressings on the wound healing process. H&E staining (Fig. 5d) revealed that epithelial cells migrated towards the deeper layers around the wound edges in both control and PPD groups, forming a “V”-shaped structure accompanied by diffuse lymphocytic infiltration, with the newly formed granulation tissue remaining unconnected. The structures of the oral mucosa were intact hierarchical in Jasland^TM^ and 0.5% PPPZ groups, but the subepithelial connective tissue was loose, and fiber arrangement was disordered. Notably, the 1% PPPZ group exhibited no inflammatory infiltration in the wound area, with a clear tissue hierarchy resembling normal mucosal structure. Similarly, Masson staining also illustrated the ordered collagen deposition and scattered vascular structure formation in 1% PPPZ treatment group, verifying the excellent wound tissue repair.

**Fig. 6:**
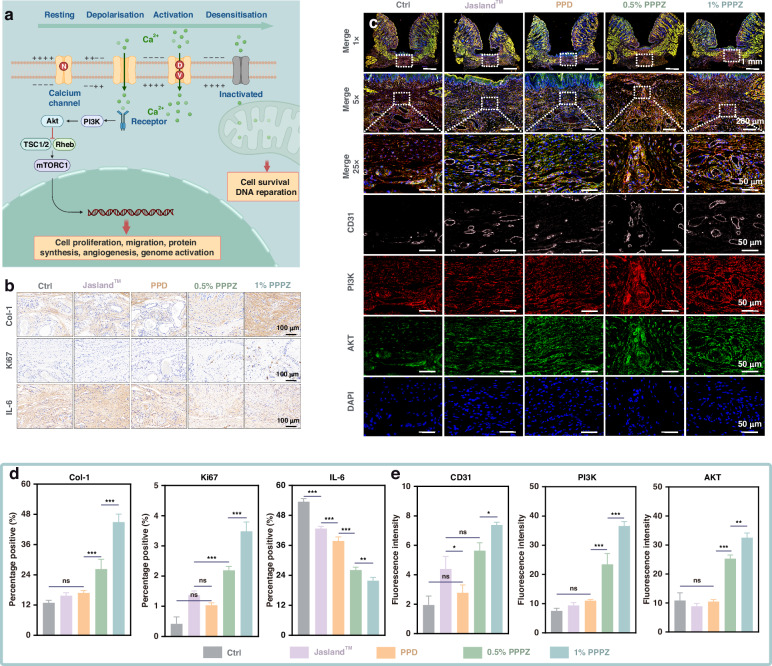
**Histological Analysis by Immunohistochemistry and Immunofluorescence.** **a** Schematic representation of piezoelectric hydrogel promoting wound healing through the PI3K/AKT signaling pathway. **b** Immunofluorescence staining of regenerated mucosal tissue labeled with Col-1, Ki67, and IL-6. Scale bars: 100 μm. **c** Immunofluorescence staining of CD31 (white), PI3K (red), and AKT (green) after wound treatment in different groups. Scale bars: 50 μm. **d** Quantitative analysis of Col-1, Ki67, and IL-6 positive area, *n* = 4. **e** Quantitative analysis of the relative fluorescence intensity of CD31, PI3K, and AKT, *n* = 3. *p < 0.05, **p < 0.01, and ***p < 0.001 indicated statistical difference and ns denoted no significant variation

During the in vivo wound healing process, the deposition of collagen, cell proliferation, and inflammatory response are essential biological events for accelerating structural repair and biological function reconstruction. Hence, we analyzed the expression of Col-1 (Type I Collagen), Ki67 (a cell proliferation marker), and IL-6 (an inflammatory response marker) in the oral buccal mucosa wound area by immunohistochemical staining. As shown in Fig. 6b, d, the 0.5% PPPZ and 1% PPPZ groups exhibit higher positive area percentages for Col-1 and Ki67 compared to the other groups, while 1% PPPZ group still displayed the most optimal performance. Meanwhile, the expression of IL-6 was the lowest in 1% PPPZ group, with a statistically significant difference. These experimental findings suggest that the promoting process of wound healing by 1% PPPZ contains not only structural protein deposition, but also regulation of cellular proliferation activities and inflammatory responses.

Previous results have proven that hydrogels can alter Ca^2+^ levels in cellular experiments. Recent research suggests that intracellular Ca^2+^ binding to receptors would trigger the activation of the intracellular PI3K/AKT signaling cascade, resulting in various biological phenotypic changes, including cell proliferation and angiogenesis. To further elucidate the molecular mechanisms and validate the action pathway, we employed multi-label immunofluorescence staining to analyze the activation status of PI3K and AKT and their impact on the angiogenic factor CD31. As shown in Fig. 6a, c, e, the signaling axis was activated during the healing process, with both PPPZ groups exhibiting significantly stronger expressions of PI3K (red fluorescence) and AKT (green fluorescence) compared to the control, Jasland^TM^, and PPD groups. Additionally, the expression of the angiogenic factor CD31 (white fluorescence) displayed the same situation as PI3K and AKT activation, indicating the promoting neovascularization effect by PPPZ. These findings support the previous hypothesis that PPPZ could facilitate cell proliferation and angiogenesis by activating the PI3K/AKT signalling pathway, thus providing molecular evidence for their application in enhancing the oral wound healing process. Furthermore, the biosafety of ZnO particles in vivo was evaluated through histological analysis of major organs in rats (Fig. S26). The results revealed no abnormal changes in the morphology and microstructure of the vital organs in all samples, further confirming the outstanding biosafety of this material in vivo.

## Discussion

In this study, based on the concept of “integrated construction”, we ingeniously fabricated a novel dual-step photo-induced method to fabricate the PPPZ hydrogels dressings for endogenous oral wound healing. The photo-induced method contains two types of mechanism: O_V_-induced photocatalysis and PET-induced photopolymerization, in which the binding of O_V_-rich ZnO and PDA promote the continuous effect.

The photo-induced method utilized the overlooked optical properties of ZnO in wound dressings with the introduction of O_V_ and consturction of interfaces, achieving the even-distributed in-situ formation of PDA in a dual-network hydrogel template under indoor environment. Compared to traditional synthesis of PDA-based hydrogels, this method is capable of synthesizing high-performance hydrogels in a continuous process, which does not require conditions such as pH-control or UV catalysis. The as-prepared PPPZ exhibits not only excellent mechanical properties such as wet adhesion and self-healing abilities but also outstanding in vitro biocompatibility. At the same time, we need to point out that this method is related to the annealing temperature and the ambient temperature during hydrogel preparation. Excessively low temperatures may limit the effective occurrence of this method, which requires further research in the future. Similarly, if there are materials within the components that coordinate with DA monomers, it may also limit the effectiveness of the method. Therefore, we recommend carefully considering the coordination between components when designing the structural composition of the hydrogel.

Due to the piezoelectric characteristic of ZnO, the PPPZ could produce a microelectric field around wounds under external mechanical stimulation, which enhances the antibacterial performance and facilitates cell proliferation and migration of *HOFs*. In vivo experiments further validate that PPPZ markedly elevate intracellular Ca^2+^ levels in *HOFs*, subsequently activating signaling pathways related to cell proliferation and angiogenesis, thereby facilitating the process of wound healing. The PPPZ hydrogel displays excellent comprehensive performance through an extremely simple preparation method, which shows immense potential for further application, and provides new ideas for the design of oral dressings.

## Materials and methods

### Materials

Polyvinyl alcohol (PVA, 54.0–66.0 mPa s^−1^ viscosity), Poly(ethylene glycol) diacrylate (PEGDA, molecular weight ~600), Dopamine HCl (DA), Zinc acetate dihydrate (Zn(CH_3_COOH)_2_·2H_2_O) sodium hydroxide (NaOH) were purchased from Sigma-Aldrich (Shanghai, China). Phosphate buffer solution (PBS) and 10% neutral formalin were purchased from Servicebio company (Shanghai, China). H&E and Masson’s trichrome Staining Kit were purchased from Beyotime Biological Company (Shanghai, China). Primary antibodies against Col-1, Ki67 and IL-6 monoclonal antibodies were purchased from Proteintech Inc. (Wuhan, China). Tissue immunofluorescence primary antibodies, CD31, PI3K, and AKT were purchased from Abcam Inc.

### Synthesis processes of ZnO and ZnO/PDA

1.552 g Zn(CH_3_COOH)_2_·2H_2_O was dissolved in 40 mL deionized water. Then, 3.2 g sodium hydroxide (NaOH) was added to the solution and stirred at room temperature for 1 h. These solutions were transferred into a 100 mL autoclave and kept at 100 °C for 24 h in an oven to form a ZnO nano-crystalline. After the reaction, the white precipitate was centrifuged and washed 3 times alternately with water and ethanol via repeated re-dispersion and filtration. The sample was then dried at 80 °C for 10 h. The products were named as ZnO.

The as-prepared ZnO was annealed at 400 °C in Ar atmosphere for 2 h, which was named as ZnO-D while which annealed at 800 °C in O_2_ atmosphere for 2 h was named as ZnO-O.

0.04 g DA was dissolved in 10 mL deionized water, and 0.002 g ZnO, ZnO-D and ZnO-O were added into three bottles of solutions, respectively. After 30 min stirring, the color changes were analyzed by digital photos. At this point, some ZnO/PDA has formed, but there is still a significant amount of unreacted DA remaining in the solutions. Then the solutions were centrifuged and washed 3 times alternately with water and ethanol. The products were dried at 80 °C for 10 h. We conducted tests for the catalytic formation of ZnO-PDA at various temperatures, and specific information can be found in the supplemental materials (Fig. S18). Briefly, this effect can be initiated at room temperature of 25 °C, while the catalytic effect is less pronounced at 15 °C.

### Preparation process of dual-network hydrogels

The synthesis of ZnO/PDA followed the method above. Briefly, 0.04 g DA was dissolved in 10 mL deionized water, and an appropriate amount of ZnO was added to a solution, stirred at a speed of 600 rpm for 30 minutes at room temperature, and the solution would turn notably violet-red in colour. Then 0.05 g PEGDA and 1.0 g PVA were then added to solution after 30 min stirring. The mixtures were keeping stirring for 60 min with 90 °C water-bath heating. The resulting products treated with ultrasonic were then poured into molds and frozen at −20 °C for 24 h. After that, the molds were left at room temperature to thaw, while the just-thawed hydrogel were named as PPDZ. The PPDZ were placed at a table indoor with no extra operation, after 12 h, the hydrogels were named as PPPZ. The PPPZ hydrogels were numbered based on the mass percentage of ZnO to PVA. Adding 0.005 g of ZnO would be labeled as 0.5% PPPZ, and adding 0.01 g ZnO would be labeled as 1% PPPZ. Since increasing the mass of ZnO further would result in insufficient dissolution, the experiment only synthesized and compared samples with 0.5% PPPZ and 1% PPPZ.

As comparation sample, the PVA, PPZ and PPD were synthesized by previous methods with different components. For the preparation of PVA hydrogel, 1.0 g of PVA is added to 10 mL of deionized water and stirred at 90 °C for 60 minutes. The resulting solution is then subjected to ultrasonic treatment to remove internal bubbles, followed by freezing at −20 °C for 24 hours. The synthesis of PPD and PPZ is similar to that of PPPZ, except for the addition of ZnO and DA, respectively, during the hydrogel construction process.

### Characterizations

The annealing process is achieved through BEQ BTF-1200C-III-S-SL CVD instrument. Before initiating the temperature rise, we first evacuated the tube furnace for 30 minutes. Then, we continuously introduce the corresponding gas into the quartz tube at a flow rate of 80 scm for 1 hour to remove residual air. Following this, we increase the temperature at a rate of 10 °C per minute. After annealing, we wait for the tube furnace to cool naturally to room temperature before retrieving the samples.

Rigaku SmartLab SE system was used to characterize XRD spectra with Cu kα line (*λ* ≈ 1.54 Å). The target for XRD testing is copper, with a scanning range of 10–80° and a scanning rate of 2° per minute.

UV-Visible absorption spectra were measured by Shimadzu UV-3600 and Thermo Evolution 350. The former is used to measure the transmittance of powder samples, while the latter is used to measure the transmittance of hydrogel films.

The FTIR curves were measured by Thermo Fisher Scientific Nicolet iS20. The wavenumber range is from 400–4000 cm^−1^. ATR (Attenuated Total Reflection) mode is used for testing liquids and hydrogels, while conventional powder pressing is used for testing powders.

SEM image and Energy-disperse X-ray spectroscopy (EDS) data were obtained from ZEISS GeminiSEM 300. A trace amount of sample is directly adhered to conductive adhesive. The Quorum SC7620 sputtering coater is then used to sputter gold for 45 seconds, with a sputtering current of 10 mA. Subsequently, the sample’s morphology and EDS mapping are captured using the ZEISS GeminiSEM 300 scanning electron microscope. The acceleration voltage for morphology imaging is set to 3 kV, while for EDS mapping, the acceleration voltage is 15 kV, with the detector being the SE2 secondary electron detector.

XPS spectra were collected on Thermo Fisher Nexsa. We cut a sample of appropriate size (5*5 mm in length and width) and attach it to the sample holder. Place the sample into the sample chamber of the instrument. When the pressure in the sample chamber is less than 2.0 × 10^−7 ^mbar, transfer the sample into the analysis chamber. Set the beam spot size to 300μm, the operating voltage to 12 kV, and the filament current to 6 mA. For survey scan, set the pass energy to 150 eV with a step size of 1 eV; for narrow scan, set the pass energy to 60 eV with a step size of 0.1 eV.

Bruker EMX plus was used to collect EPR spectra. In the oxygen vacancy test, the experimental temperature is set to 77 K, and the test is conducted after illuminating the sample for 10 minutes. In the hydroxyl radical-related tests, we conducted measurements on hydrogel samples both before and after exposing them to xenon lamp light for 30 minutes.

The BET results were recorded by Micromeritics ASAP 2460. Using the standard degassing station of Micromeritics, the samples are pretreated under vacuum at 120 °C for 8 hours. Then, the 4-station fully automatic specific surface area analyzer model APSP 2460 from Micromeritics (USA) is used to conduct nitrogen adsorption-desorption tests on the samples at 77 K using liquid nitrogen. After the instrument completes the analysis, an isothermal adsorption-desorption curve is obtained. The total specific surface area of the material is then calculated using the BET method.

TEM characterizations were performed on a Talos F200X, FEI microscope. We subjected the sample to ultrasonic treatment in water for ten minutes and prepared the sample using a copper microgrid for analysis. The TEM was operated at a voltage of 200 kV with a magnification of 1.5 million times. The EDS model is Ultim Max 80.

The contact angle of hydrogels was measured by Sindin SDC 350KS. The software could achieve precise numerical readings of the contact angle based on the 3D morphology method.

The PL and Raman spectra were recorded under 532-nm excitation by a Confocal Laser Raman Spectrometer (Witec Alpha 3000 R). The CCD model is Andor DV420_OE. All tests were conducted under a ×50 objective lens with the central wavelength set to 2000 cm^−1^. The grating model is Acton HRS-300, which was set to G2 during the experiment, and the laser power was set to 10 mW.

The tensile tests were performed on a ZQ-990L electronic universal testing machine. Before performing the tensile test, the corresponding gauge length and contact area are determined, and the tensile rate is set to 20.0 mm per minute.

### DFT calculation

All simulations were carried out using spin-polarized methods as implemented in the QUICKSTEP code of the CP2K 2024 package, which is based on density functional theory. The general gradient approximation (GGA) parametrized by Perdew, Burke, and Ernzerhof was used as the exchange-correlation functional. The Kohn–Sham (KS) equations are solved according to the Gaussian and plane wave (GPW) formalism. Grimme’s DFT-D3 correction was adopted to describe the weak van der Waals interaction. The GPW uses Goedecker–Teter–Hutter pseudo potentials to describe the interactions between core and valence electrons, while the valence electron density is represented in terms of Gaussian type orbital basis set functions. In particular, we use DZVP-MOLOPT-SR-GTH basis sets for geometry optimization and TZVP-MOLOPT-SR-GTH basis sets for static calculations, and the Brillouin zone integration was sampled using a Monkhorst-Pack special k-point mesh with a resolution of 2π*0.04 was applied. The convergence criterion for the maximum force was set as 5 × 10^−4^ atomic units. The auxiliary PW basis set, which is needed for the efficient solution of Poisson’s equation in reciprocal space, is truncated at 500 Ry. All Electronic structure and wave function analyses were conducted using Multiwfn software.

### Cytotoxicity assay in vitro

In this study, *HOFs* were utilized to evaluate the cytotoxicity of different piezoelectric hydrogel systems. First, the hydrogel materials were thoroughly disinfected. The material extracts were prepared using Dulbecco’s Modified Eagle Medium complete culture medium containing 10% fetal bovine serum and 1% penicillin-streptomycin at a mass ratio of 1:10. Subsequently, *HOFs* were seeded into 24-well plates at a density of 5 × 10^4^ cells/well and cultured with the aforementioned extract-containing culture medium, with four wells per group. Simultaneously, a normal culture group was set up as a control. On days 1 and 2, cells were stained using the Live-Dead Cytotoxicity Assay Kit (Beyotime, China) and observed under a fluorescence microscope (Leica DMi8, Germany). Live cells were labelled with Calcein-AM, displaying green fluorescence, while dead cells were labelled with propidium iodide (PI), displaying red fluorescence. Photographs were captured by an inverted fluorescence microscope, and the proportion of live and dead cells was statistically analyzed using Image J software.

### Biocompatibility assessment in vivo

This study employed a subcutaneous implantation model in rats to evaluate the in vivo biocompatibility and biodegradability of the hydrogel material. All experimental animals were provided by Shanghai Shengchang Biotechnology Co., Ltd., and all animal experiments were approved by the Animal Ethics and Use Committee of Shanghai Shengchang Biotechnology Co., Ltd. (Ethics No. 2024-07-KOYY-WXL-079). 6 male Sprague-Dawley rats (8 weeks old, weighing 200–220 g) were selected. Under anesthesia with 3% pentobarbital sodium (30 mg/kg), the surgical site was prepared and disinfected. A 1 cm longitudinal incision was made on the dorsal skin, followed by blunt dissection to create a subcutaneous pocket under sterile conditions. The 1% PPPZ hydrogel material was implanted into the pocket, and the wound was sutured. The animals were euthanized on postoperative days 4, 7, 14, and 28. Wound healing and material degradation were photographed and recorded. Skin tissues surrounding the material were collected for histological analysis to assess the material’s in vivo safety.

### Cell attachment experiment

A 24-well culture dish was coated with 1% PPZP piezoelectric hydrogel. The hydrogel was soaked in cell culture medium for 12 hours and then washed with PBS three times. HOFs and HUVECs were seeded onto the hydrogel surface at a density of 1 × 10^5^ cells/well. After 24 hours of incubation at 37 °C and 5% CO_2_, samples were fixed with 2.5% glutaraldehyde (Servicebio, China) for 2 hours. Subsequently, cells were washed with PBS, dehydrated with a gradient of 30%, 50%, 70%, 90%, and 100% ethanol, dried in a fume hood, sputter-coated with gold, and observed using SEM to assess cell morphology.

### Hemolytic activity

The blood compatibility of hydrogels was verified through hemolysis experiments. Briefly, 5 mL of fresh blood from Sprague-Dawley (SD) rats was collected and mixed in an anticoagulant tube containing ethylenediaminetetraacetic acid. After standing for 5 minutes, a small amount of blood clots was removed. Then, 10 mL of isotonic saline was added, and the mixture was centrifuged at 1200 rpm for 10 minutes. The red blood cell suspension was repeatedly washed until the supernatant was clear and transparent, and finally resuspended to a 5% (v/v) concentration. Subsequently, 0.6 mL of hydrogel extract, distilled water (negative control), and 0.9% saline (positive control) were mixed with 0.6 ml of the red blood cell suspension and incubated at 37 °C for 1 hour to observe hemolysis. Next, 100 μL of the supernatant from each group was added to a 96-well plate, and the optical density (OD) was measured at a wavelength of 540 nm using a microplate reader. Finally, the hemolysis rate was calculated using the following formula:

Hemolysis rate (%) = (OD Sample – OD Control)/(OD Positive Control–OD Negative Control) × 100%

### Antibacterial activities

The antibacterial properties of the hydrogels were investigated using *Staphylococcus aureus* (*S. aureus* ATCC 6538) and *E. coli* (ATCC 11229). First, the hydrogels were cut into circular samples with a diameter of 6 mm and a thickness of 2 mm using a punch. Then, 100 μL of bacterial suspensions (10^7^ CFU/mL) of *S. aureus* and *E. coli* were evenly spread on agar plates. The hydrogel samples and drug sensitivity disks (positive control) were placed on the agar plates, and the plates were incubated for 18 hours. During incubation, the hydrogel groups received US stimulation (1 W/cm^2^, 1 MHz) for 10 minutes. After incubation, the diameters of the inhibition zones were recorded.

Additionally, the LIVE/DEAD™ BacLight™ Bacterial Viability Kit (Invitrogen™ L-7012, USA) was used to investigate the bactericidal effect of hydrogels against *Streptococcus mutans* (*S. mutans* ATCC 25175). The hydrogel samples were co-cultured with 1 mL of *S. mutans* suspension for 12 hours, with 50 μL of saline serving as a blank control. Bacteria were stained according to the manufacturer’s instructions, and 5 μL of the bacterial staining solution was placed on a slide for observation under a fluorescence microscope. Bacteria with intact cell membranes were stained green by SYTO 9, while bacteria with damaged membranes were stained red by PI. The bactericidal rate was evaluated based on the ratio of live to dead bacteria.

### EdU cell proliferation assay

An in vitro piezoelectric hydrogel cell stimulation chamber was constructed using Transwell inserts, as shown in Fig. 4e. *HOFs* were seeded onto the bottom of 12-well plates at a density of 5 × 10^4^ cells/well. The hydrogel was placed in the upper chamber of the Transwell insert, and 1 ml of culture medium was added. During culturing, the hydrogel was activated with ultrasound (10 min/day, 1 W/cm^2^, 1 MHz). After 1 day of intervention, EdU staining was performed according to the manufacturer’s instructions for the EdU staining kit (Beyotime, China). Briefly, *HOFs* were co-incubated with EdU for 3 hours, fixed with 4% paraformaldehyde for 15 minutes, permeabilized with 0.3% Triton X-100 for 15 minutes, and then incubated with the click reaction mixture from the kit at room temperature in the dark for 30 minutes. Finally, the cells were stained with Hoechst and imaged using an inverted optical microscope. EdU-positive cells were counted using Image J software.

### Scratch assay

*HOFs* were seeded into 6-well plates at a density of 2 × 10^5^ cells/well. When the cell density reached 90%, a straight line was scratched across the bottom of the plate using a 200 μL pipette tip to create a cell-free area. Detached cells were removed with PBS. The cells were then cultured in a medium containing 1% serum, and the scratch area was recorded using an inverted optical microscope (Leica DMIL, Germany) at 0 and 24 hours.

### Experiments for the detection of intracellular calcium ions

The intracellular Ca^2+^ level was measured using the Fluo-4 AM Calcium Assay Kit (Beyotime, China). HOFs were seeded into 24-well plates at a density of 5 × 10^4^ cells/well and cultured for 12 hours to allow cell attachment. Cells were then treated according to the groups described in EdU test. Following the manufacturer’s instructions, Fluo-4 AM staining solution was prepared and added to each well (250 μL per well). Cells were incubated at 37 °C in the dark for 30 minutes, washed three times with PBS, and observed under a fluorescence microscope at 488 nm. The cells were then collected and analyzed using flow cytometry.

### Tube formation assay

The tube formation assay was used to evaluate the in vitro angiogenesis capability of *HUVECs*. First, 10 μL of Matrigel scaffold (Corning, USA) was added to Ibidi slides. *HUVECs* were seeded onto the Matrigel at a density of 2 × 10^4^ cells/well and incubated at 37 °C for 8 hours. Microscopic images were captured, and the number of *HUVEC* tubes formed was quantitatively analyzed using ImageJ software.

### Assay of intracellular ROS

*HOFs* were seeded onto the bottom of 12-well plates at a density of 5 × 10^4^ cells/well. After the cells adhered to the wells, *HOFs* were loaded with the DCFH-DA probe according to the instructions provided with the ROS assay kit. Different groups of hydrogels were then used to treat the cells for 30 minutes. A normal group (untreated control) and a positive control group were included. Hoechst nuclear staining was performed, and the cells were observed under a fluorescence microscope. The remaining cells were collected, and cell viability was assessed using the CCK8 assay kit.

### Gene expression analyses

In wound healing, the activation of Ca^2+^ particle receptors triggers the classical downstream PI3K/AKT signaling pathway. To investigate this, we performed qPCR to measure the expression levels of the associated transcription factors. Total RNA was extracted from the post-intervention cells using Trizol reagent (Takara Bio, Japan). Subsequently, RNA was reverse-transcribed into cDNA using the PrimeScript™ RT reagent kit (Takara Bio). qPCR analysis was carried out using the LightCycler 480 System (Roche, Switzerland). The primer sequences used for qPCR are listed in Supplementary Table S1.

### Wound healing effect of piezoelectric hydrogel in a mucosal defect rat model

To ensure the accuracy of the experimental results, 30 male SD rats (8 weeks old, weighing 200–220 g) were randomly divided into five groups of six rats each. All experimental animals were provided by Shanghai Shengchang Biotechnology Co., Ltd., and all animal experiments were approved by the Animal Ethics and Use Committee of Shanghai Shengchang Biotechnology Co., Ltd. (Ethics No. 2024-07-KOYY-WXL-079). Under anesthesia induced by intraperitoneal injection of 3% pentobarbital sodium (30 mg/kg), the upper and lower jaws were fixed with a mouth gag, and a 5x5x4 mm wound was created on the buccal mucosa of the rats under aseptic conditions using ophthalmic scissors. The wounds were treated with PPD, 0.5% PPPZ, and 1% PPPZ piezoelectric hydrogels, while the blank control group received no treatment, and the positive control group was treated with a commercial oral wound patch (Jasland^TM^, China). Wound changes were analyzed using ImageJ software on days 1, 3, 7, 10, and 14. The wound healing rate was calculated as follows:

$${\rm{Wound\; Healing\; Rate}}( \% )=(1-{\rm{Wound\; Area\; on\; the\; Nth\; day}}/{\rm{Total\; Wound\; Area}})\times 100 \%$$

### Histological analysis

After the experiment, the rats were euthanized, and samples of the mucous membrane tissue surrounding the wound were collected. The tissues were fixed in 4% paraformaldehyde for 48 hours and then processed into 4 μm thick paraffin sections following standard procedures. H&E and Masson staining were performed using a complete staining kit (Solarbio, China) according to the manufacturer’s instructions. Additionally, immunohistochemical staining assessed the expression of repair-promoting factors (Col-1, Ki67) and the inflammatory marker IL-6 in the wound area. Briefly, the paraffin sections underwent antigen retrieval, and the primary antibodies were diluted at a ratio of 1:200 and incubated overnight at 4 °C. Subsequently, the sections were washed with PBS and incubated with secondary antibodies at room temperature. The color was developed using a chromogenic substrate, and the nuclei were stained with hematoxylin. Finally, the sections were dehydrated using a gradient ethanol series and mounted with neutral gum. Multiple immunofluorescence staining was employed to analyze the expression of the PI3K/AKT signaling pathway and the angiogenesis marker CD31. This was performed according to the manufacturer’s protocol for multiple immunofluorescence staining, and the slides were mounted with an anti-fade mounting medium containing DAPI. Observations and photographs were taken using a fluorescence microscope, and the expression rates of these factors were statistically analyzed using ImageJ software.

### Statistical analysis

Statistical analysis was conducted by using GraphPad Prism 8 statistical software. Significant differences were calculated with variance tests (two-way analysis of variance (ANOVA) and one-way ANOVA), followed by Tukey’s multiple comparison tests when performing multiple comparisons between groups. Data represent the Mean ± SD of at least three replicates. *p < 0.05, **p < 0.01, and ***p < 0.001, were regarded as statistically significant. In addition, “ns” denoted no significant difference.

## Supplementary information

Supplemental Material

Supplemental Video 1

Supplemental Video 2

Supplemental Video 3

## Data Availability

All the data and methods needed to evaluate the conclusions of this work are presented in the main text. The data that support the findings of this study are available from the corresponding author upon request.
