# Supplementary material for: Dual-step photo-induced self-assembled hydrogel for endogenous oral mucosal wound healing
Source: Light Sci Appl. 2025 May 15;14:186. doi: 10.1038/s41377-025-01837-7 (PMC12078718; doi:10.1038/s41377-025-01837-7)
Supplement: Supplementary file 1 — Supplemental Material [file 41377_2025_1837_MOESM1_ESM.pdf]

Supplementary information for

Dual-step photo-induced self-assembled hydrogel for endogenous oral mucosal wound healing

Shaojun Fang[1], Qiangqiang Zhou[2, 3], Mengqi Zhou[2, 3], Changyi Li[2, 3], Huaxing Xu[2, 3], Hongyu Tang[4], Wanlu Zhang[5], Ruiqian Guo[4, 5], Xiaoling Wei[2, 3*], Rongjun Zhang[1, 4*]

[1]Department of Optical Science and Engineering, Key Laboratory of Micro and Nano Photonic Structures (MOE), Shanghai Engineering Research Center of Ultra-Precision Optical Manufacturing, School of Information Science and Technology, Fudan University, Shanghai 200433, China.

[2]Department of Endodontics, Shanghai Stomatological Hospital and School of Stomatology, Fudan University, Shanghai 20001, China

[3]Shanghai Key Laboratory of Craniomaxillofacial Development and Diseases, Shanghai Stomatological Hospital and School of Stomatology, Fudan University, Shanghai 20001, China

[4]Academy for Engineering and Technology, Fudan University, Shanghai 200433, China

[5] Institute for Electric Light Sources, Fudan University, Shanghai 200433, China

[*]Corresponding authors: Xiaoling Wei (xiaoling_wei@fudan.edu.cn), Rongjun Zhang (rjzhang@fudan.edu.cn).

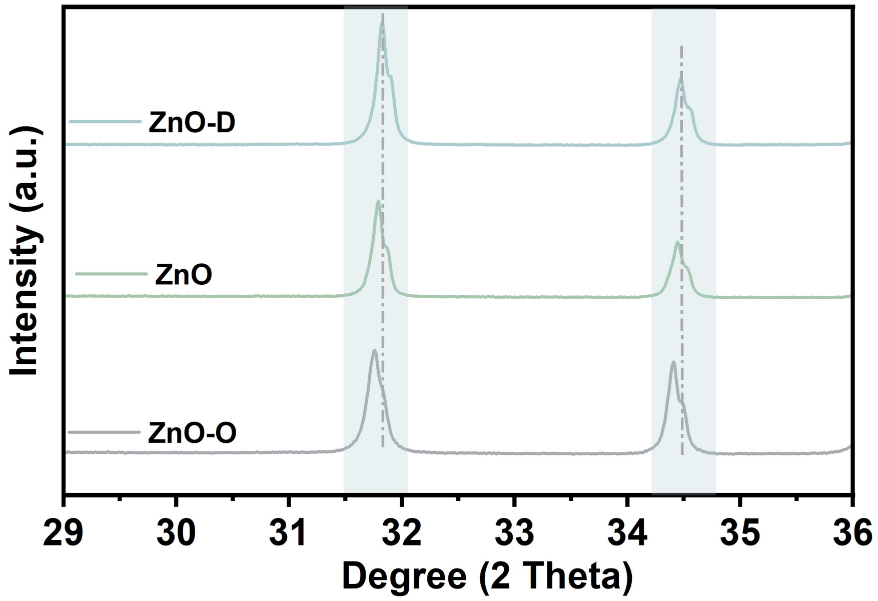

Fig. S1 XRD partial spectra of ZnO-D, ZnO and ZnO-O

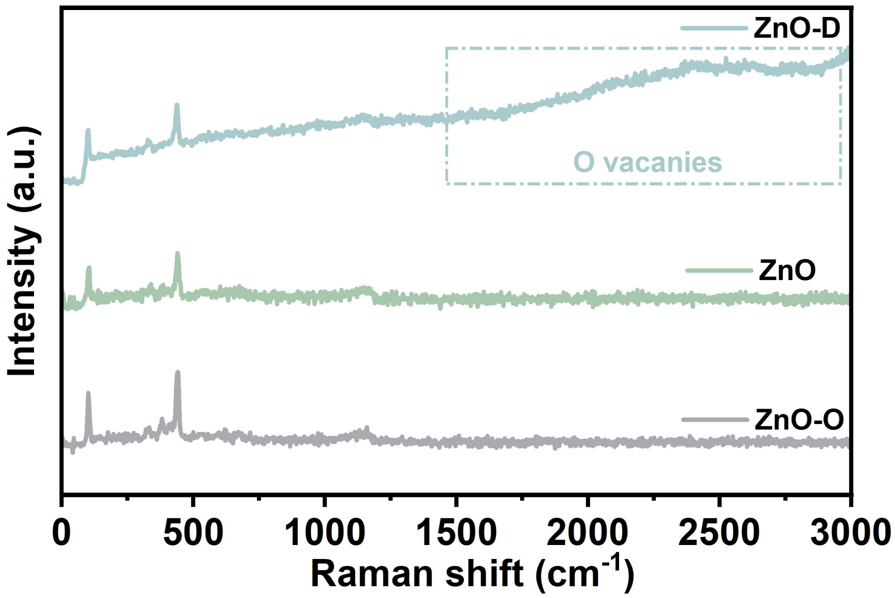

Fig. S2 Raman spectra of ZnO-D, ZnO and ZnO-O

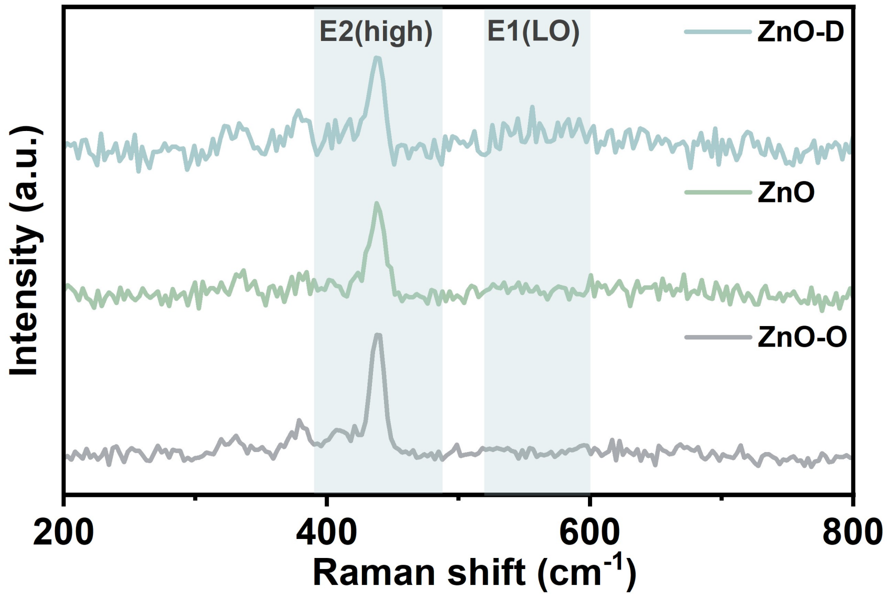

Fig. S3 Raman partial spectra of ZnO-D, ZnO and ZnO-O

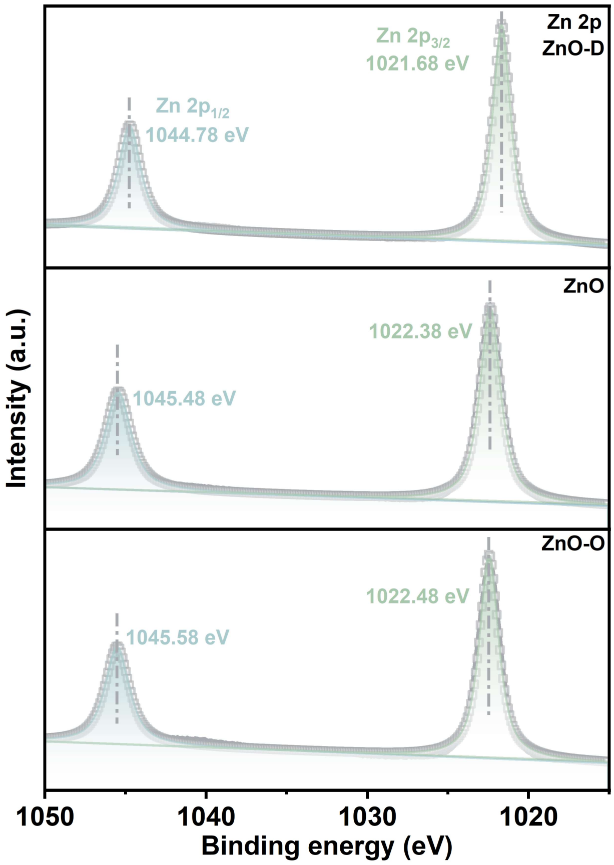

Fig. S4 Zn 2p XPS spectra of ZnO-D, ZnO and ZnO-O

Due to the presence of O_V, the attraction ability of O atoms for electrons around Zn atoms decreases, resulting in an increase in electrons surrounding Zn in the ZnO-D and a reduction in binding energy. Therefore, compared to ZnO, the binding energy of ZnO-D is lower. The situation for the ZnO-O is just the opposite.

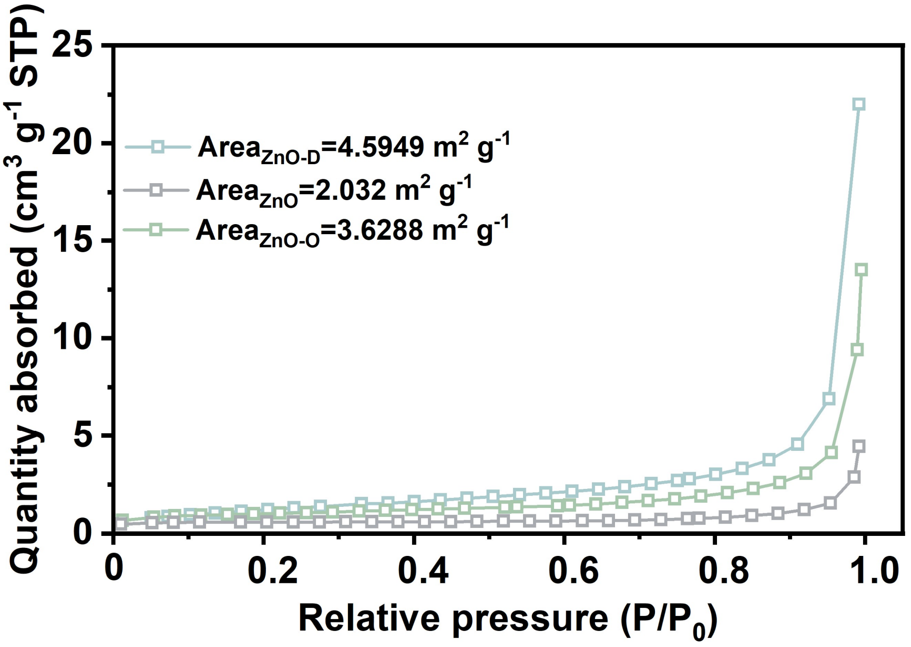

Fig. S5 BET surface areas of ZnO-D, ZnO and ZnO-O

Due to the reduction in nanorod size after annealing, the ZnO-D and ZnO-O both have a larger specific surface area compared to ZnO. However, the ZnO-O still exhibits poorer polymerization effect, indicating that specific surface area is not the decisive factor affecting the catalytic effect. Instead, the concentration of oxygen vacancies is the core determinant of catalysis.

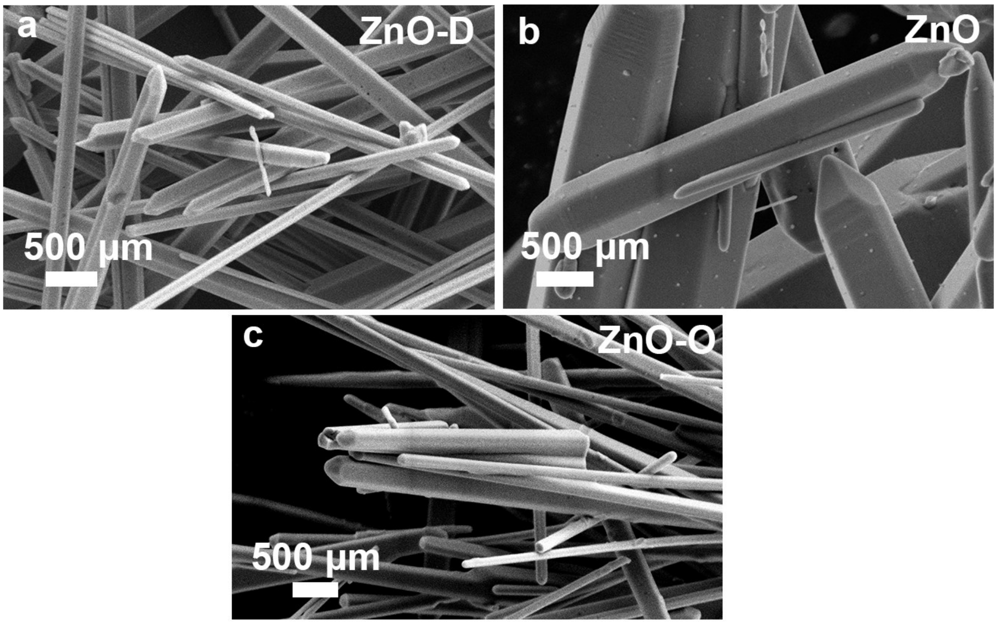

Fig. S6 SEM images of (**a**) ZnO-D, (**b**) ZnO and (**c**) ZnO-O

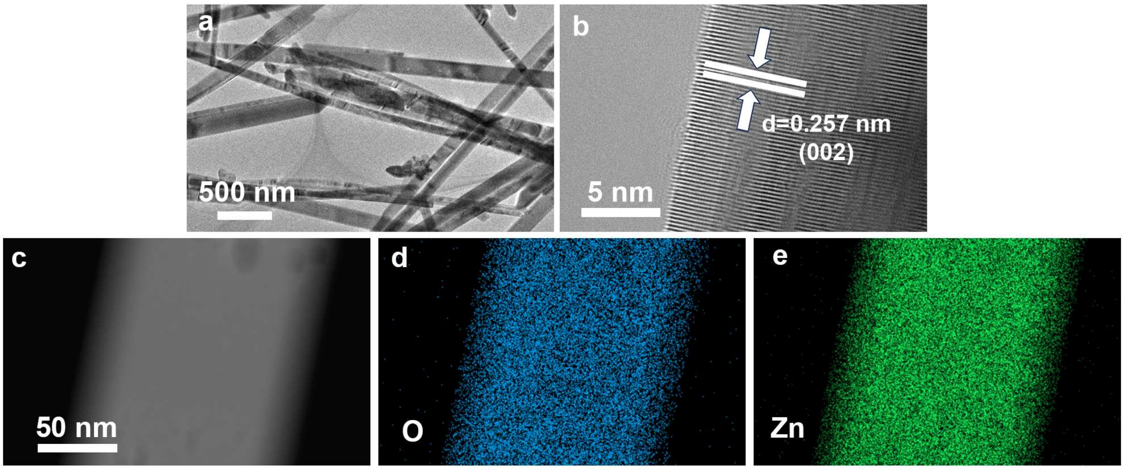

Fig. S7 a, **b** TEM images of ZnO-D, **c**-**e** EDS mapping images of ZnO-D

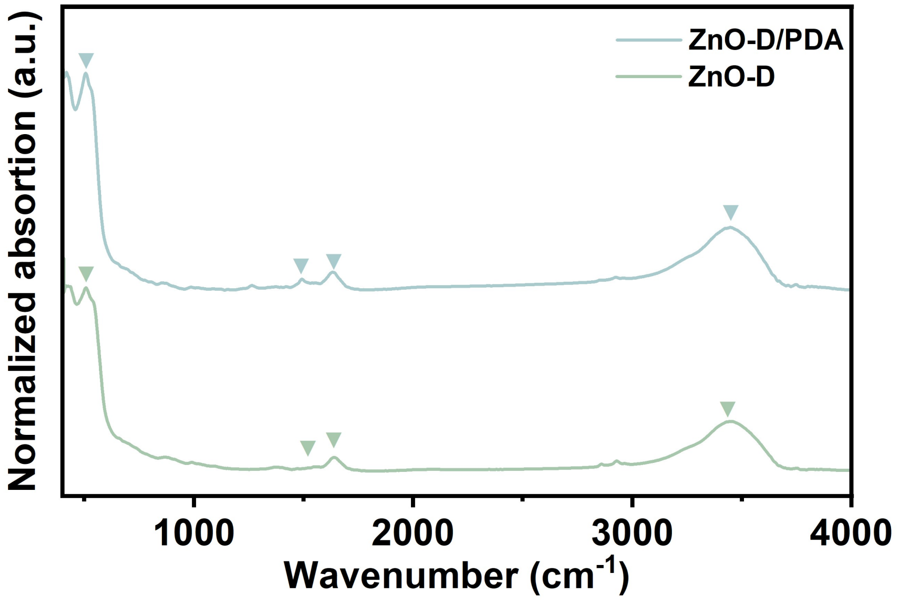

Fig. S8 FTIR spectra of ZnO-D and ZnO-D/PDA

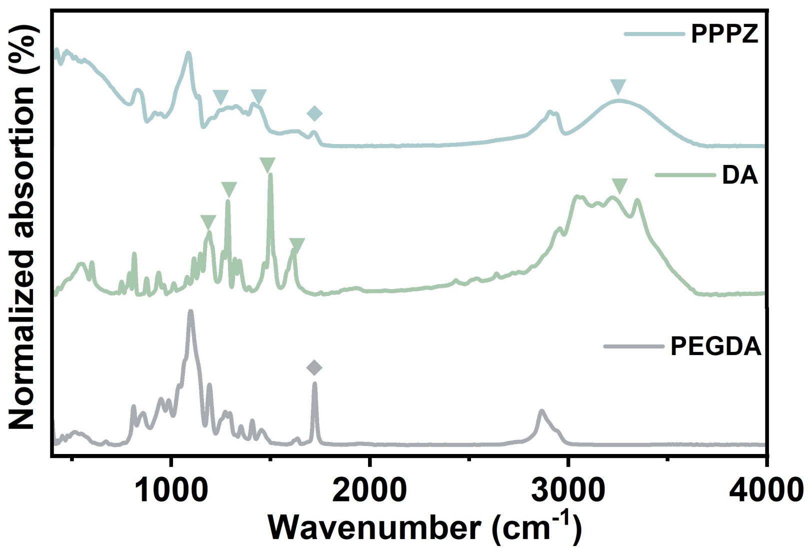

Fig. S9 FTIR spectra of PPPZ, DA and PEGDA

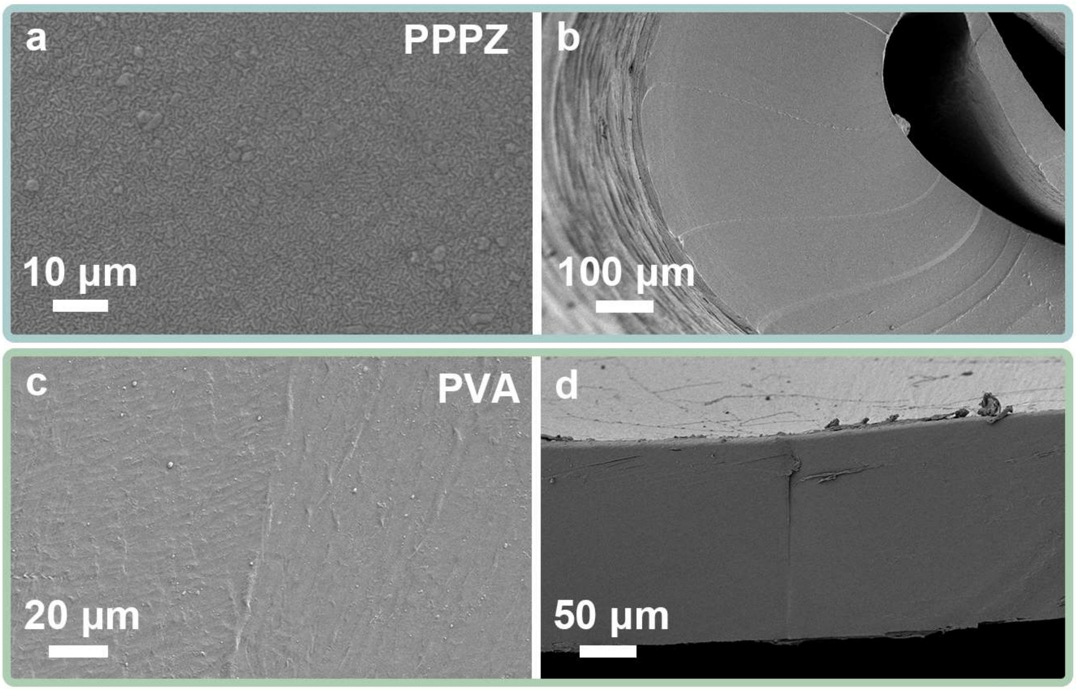

Fig. S10 SEM images of PPPZ (**a**) surface and (**b**) section; SEM images of PVA (**c**) surface and (d) section

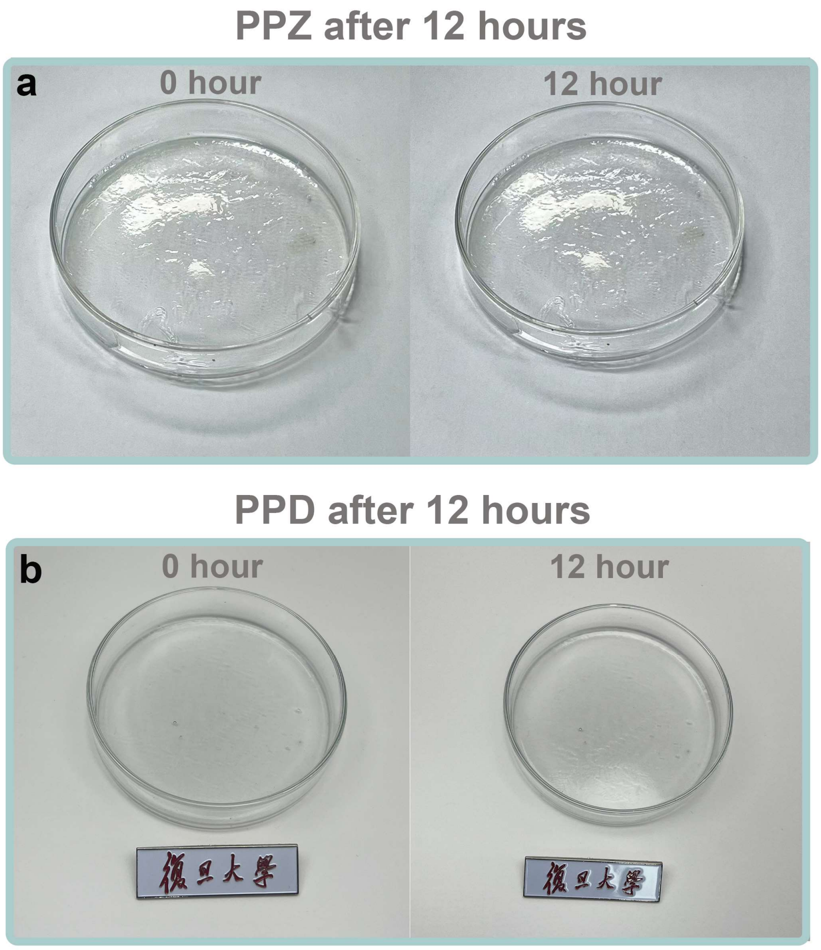

Fig. S11 The color change of PPZ and PPD after 12 hours

Due to the lack of internal photo-induced synergistic effects, neither of the PPD and PPZ exhibited any color change after being placed for 12 hours.

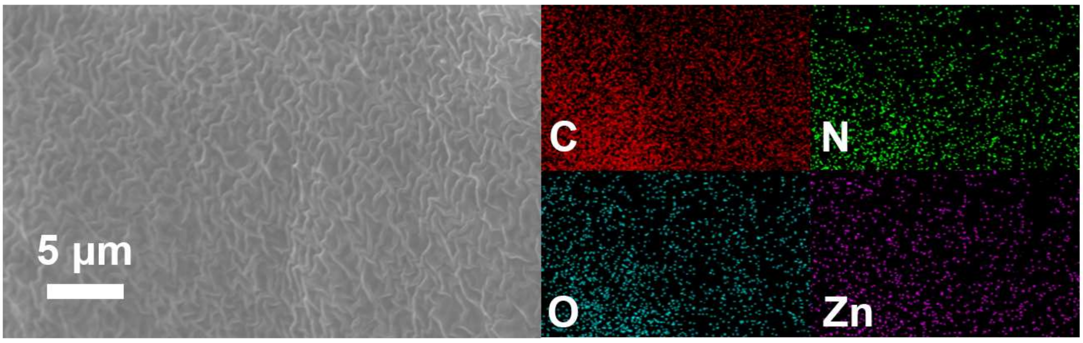

Fig. S12 The EDS mapping images of PPPZ

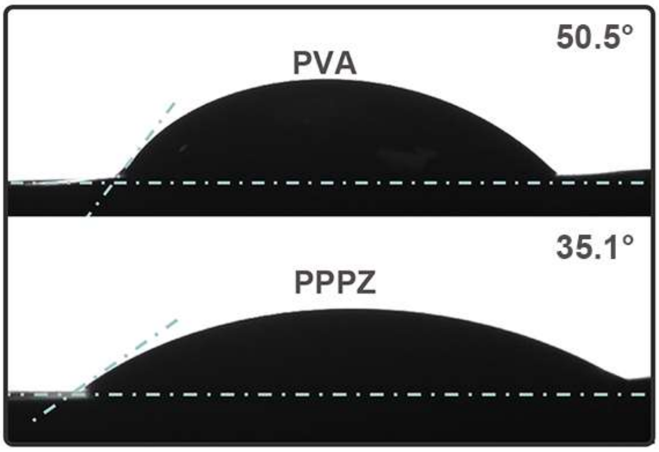

Fig. S13 The contact angles of PPPZ and PVA

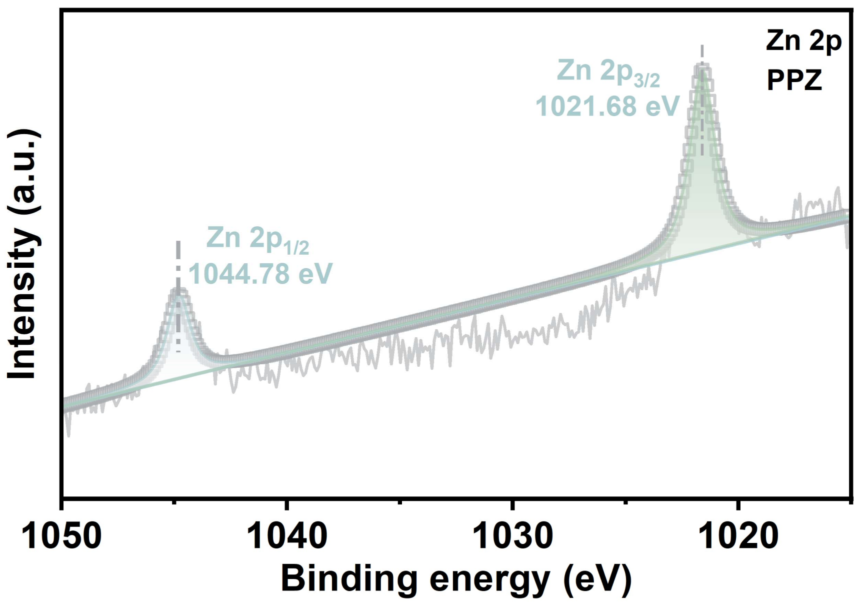

Fig. S14 Zn 2p XPS spectra of PPZ

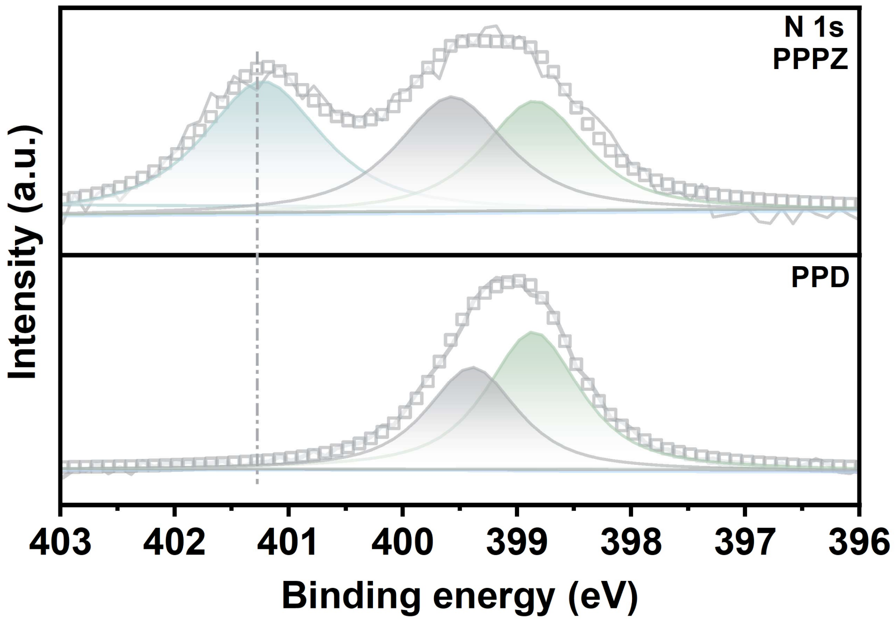

Fig. S15 N 1s XPS spectra of PPPZ and PPD

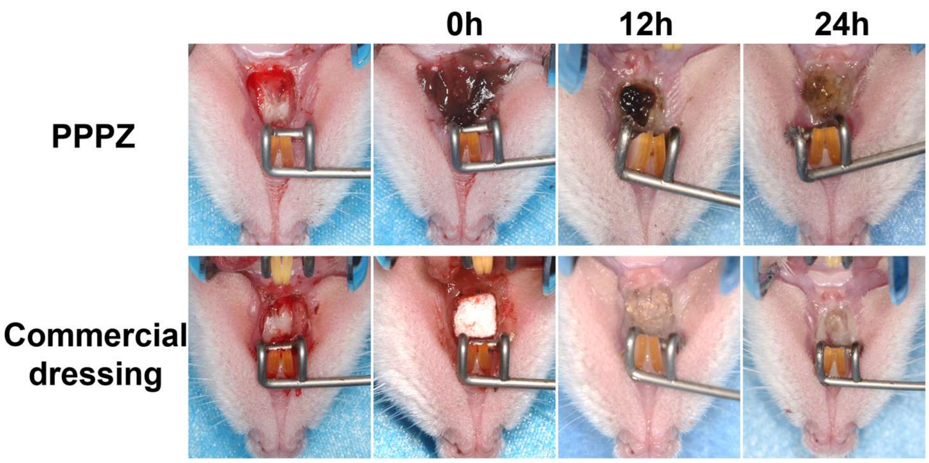

Fig. S16 Digital images of PPPZ internal adhesion after 24 hours compared to commercial dressing.

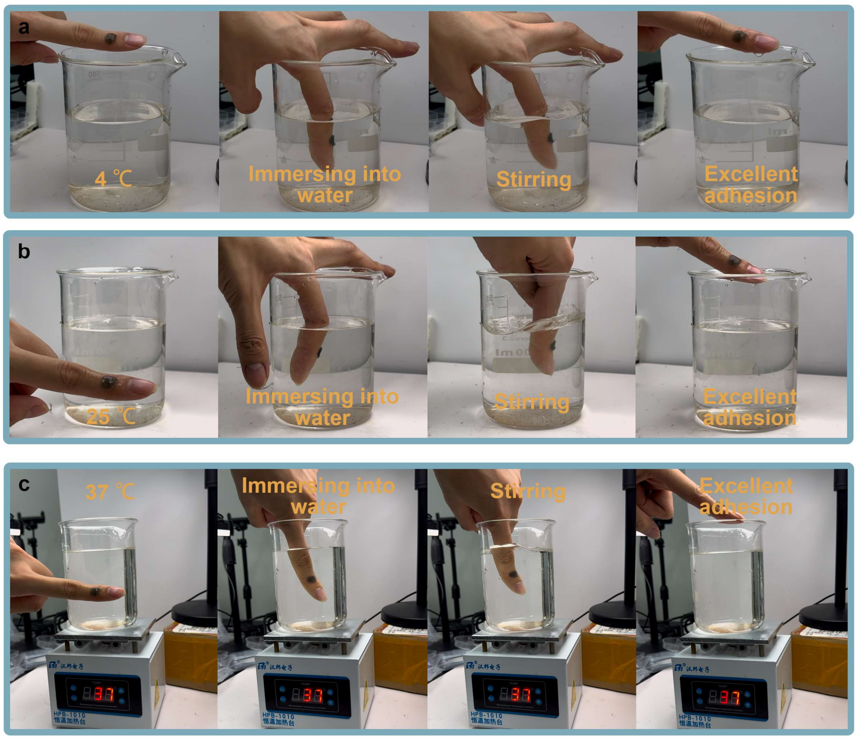

Fig. S17 Wet adhesion tests of (a) 4 ℃, (b) 25 ℃ and (c) 37 ℃

We supplement the adhesion tests at different temperatures (4 ℃, 25 ℃, and 37 ℃). The water at 4 ℃ is achieved through cooling with ice packs, while the water at 37 ℃ is reached by continuous heating on a heating platform. All tests are conducted after the relevant temperatures have been measured. As shown in Fig. S17, we adhered the same piece of PPPZ hydrogel to a finger, immersed it into water of different temperatures, and quickly stirred the finger, the PPPZ could maintain a tight fit with the finger of all temperatures. We have also uploaded the relevant Supplemental Video-3. The excellent wet adhesion performance at different temperatures further supports the rationality of the PPPZ's structural design. These will further corroborate the hydrogel's excellent wet adhesion properties.

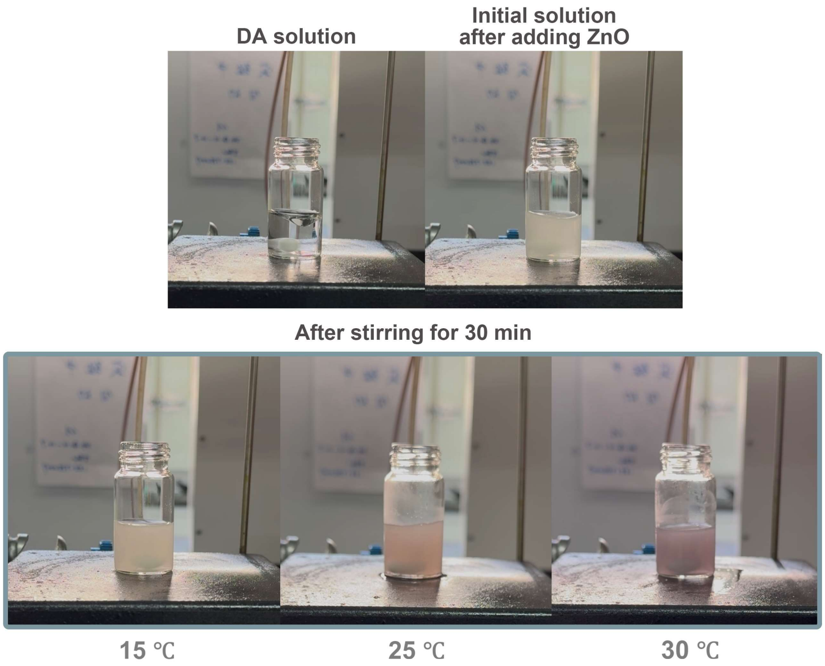

Fig. S18 Comparison of ZnO-PDA formation in the initial photo-induced step construction at different temperatures (15, 25 and 30 ℃)

We added 10mg of ZnO to three separate bottles, each containing 10mL of a solution with 40mg of DA (dopamine), and stirred the mixtures on the same stirring platform at different temperatures to observe the impact of temperature on catalytic performance. The results showed that after 30 minutes of stirring, the solution at 15 ℃ exhibited the least degree of color change. The results indicate that as the temperature increased, the catalytic performance improved. However, since the catalytic effect was also significant at 25 ℃, we chose to conduct the entire synthesis process at room temperature of 25 ℃ for the purposes of simplifying the synthesis procedure and reducing energy consumption. This discrepancy suggests that when the ambient temperature is low, mild heating may still be necessary to facilitate the occurrence of this method.

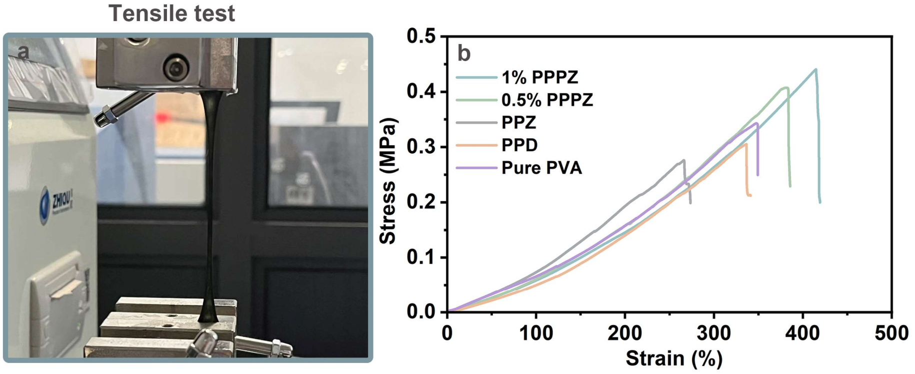

Fig. S19 Tensile test. (a) digital photograph of the test, (b) comparison of different hydrogels

As mentioned in the preface, as an inorganic material, ZnO is incompatible with polymers. This incompatibility can disrupt the stable structure of hydrogels, thereby reducing their strength. As shown in Fig. S19b, the maximum strain and stress of PPZ are both lower than those of pure PVA hydrogels. Similarly, due to the lack of effective polymerization methods, DA in PPD cannot effectively crosslink internally, and agglomeration may occur, disrupting the uniform stress distribution of the hydrogel when subjected to strain, thereby leading to a decline in performance. However, both the maximum strain and stress of PPPZ hydrogels are significantly enhanced, benefiting from the light-induced in-situ construction. The enhanced strain resistance further demonstrates the potential of PPPZ for practical applications in oral environments.

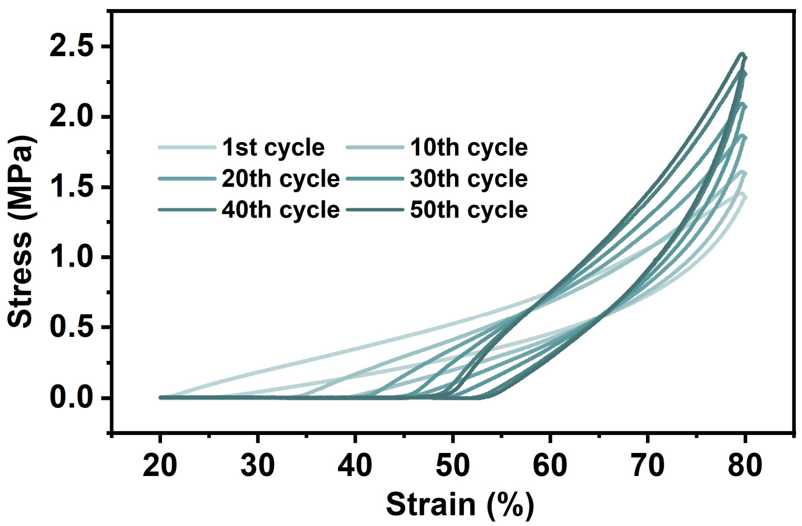

Fig. S20 Stress-strain curves of PPPZ after undergoing 80% strain tensile cycles

As the strain cycles progress, the shapes of the curves gradually turned to consistent, demonstrating the excellent strain stability of PPPZ. The results of the strain cycle test further indicate that PPPZ hydrogels can adapt to the strains caused by repeated oral movements and meet the practical usage requirements.

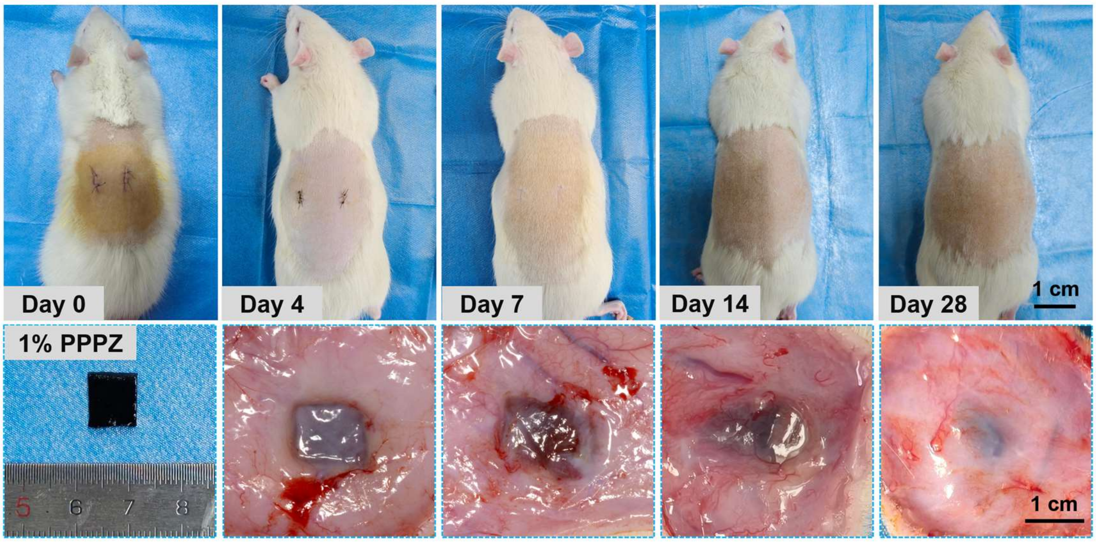

Fig. S21 Macroscopic observation results of 1% PPPZ implantation at different time points in the subcutaneous tissue of SD rats.

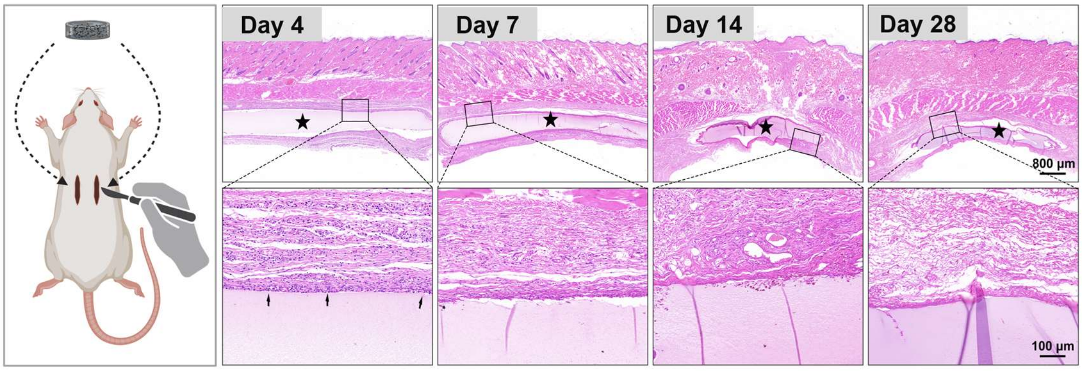

Fig. S22 Histological analysis results of 1% PPPZ implantation at different time points in the subcutaneous tissue of SD rats. The black asterisk represents 1% PPPZ, and the black arrow points to inflammatory cells.

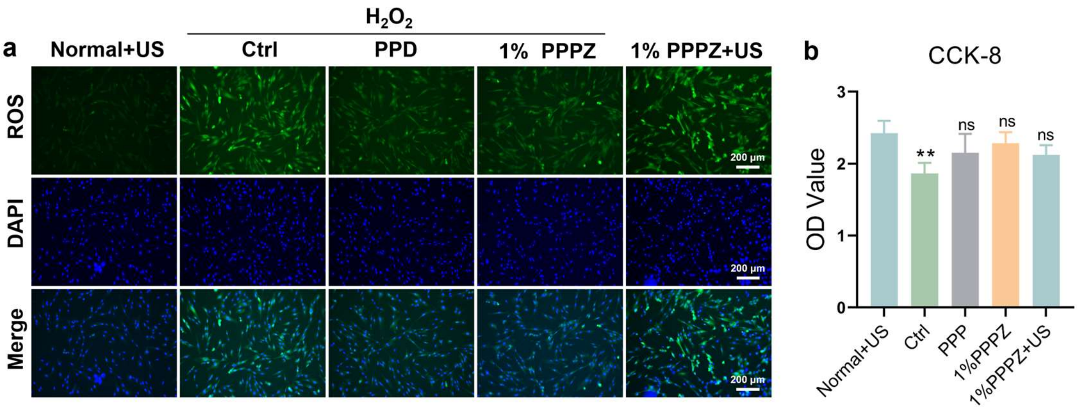

Fig. S23 a The effects of hydrogels on ROS production *in vitro* across different groups. Scale bars: 200 μm. **b** Quantitative analysis of CCK-8

We examined the effects of PPD and 1% PPPZ on intracellular ROS under different conditions using an ROS assay kit. The results showed that in the H_2O_2-induced cellular oxidation model, the presence of PPD had the ability to scavenge ROS to some extent (Fig. S23a). However, the microcurrents generated by ultrasound irradiation of 1% PPPZ could also produce a certain amount of ROS, but it did not affect subsequent cell growth (Fig. S23b).

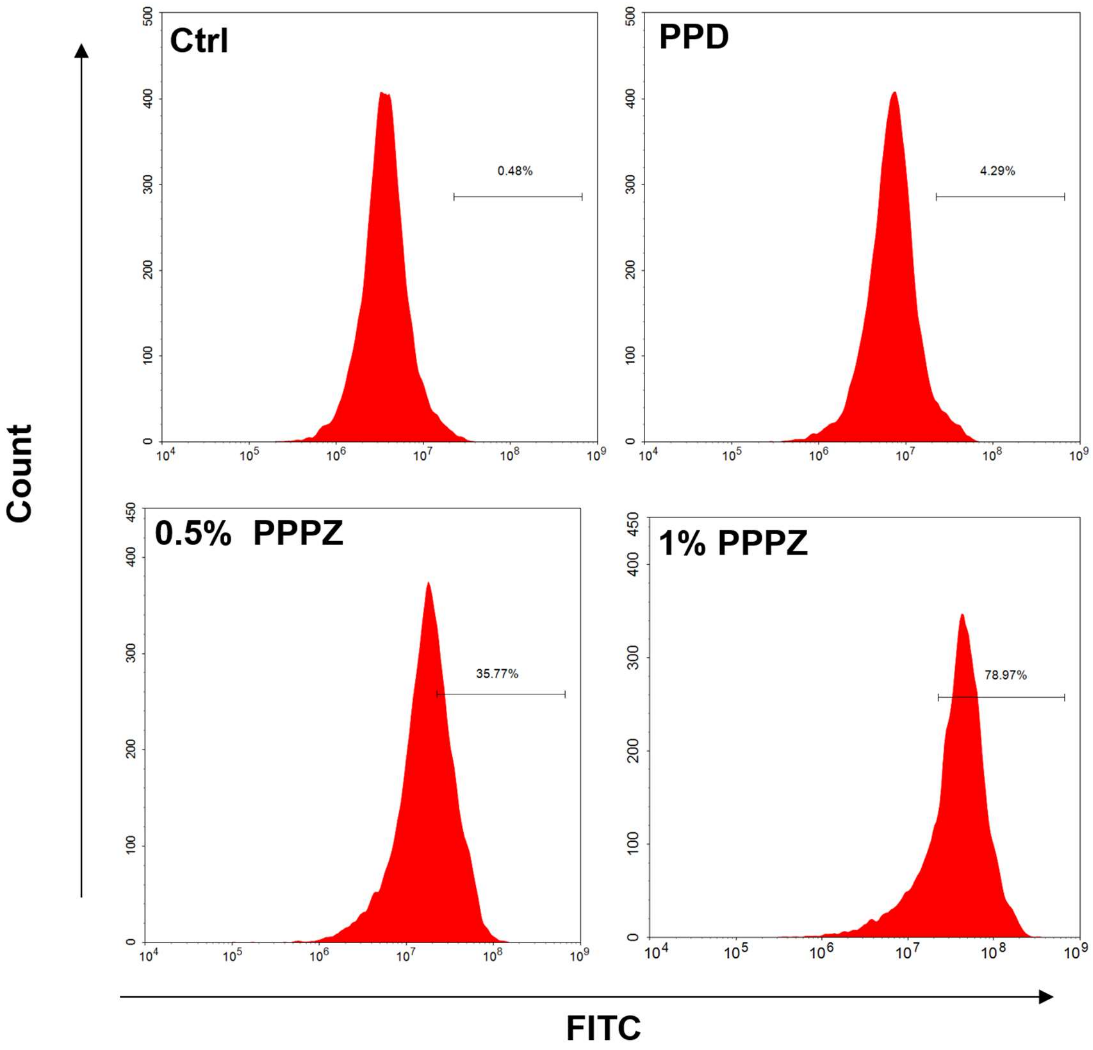

Fig. S24 Flow cytometry analysis of changes in intracellular calcium levels after treatment with different hydrogels

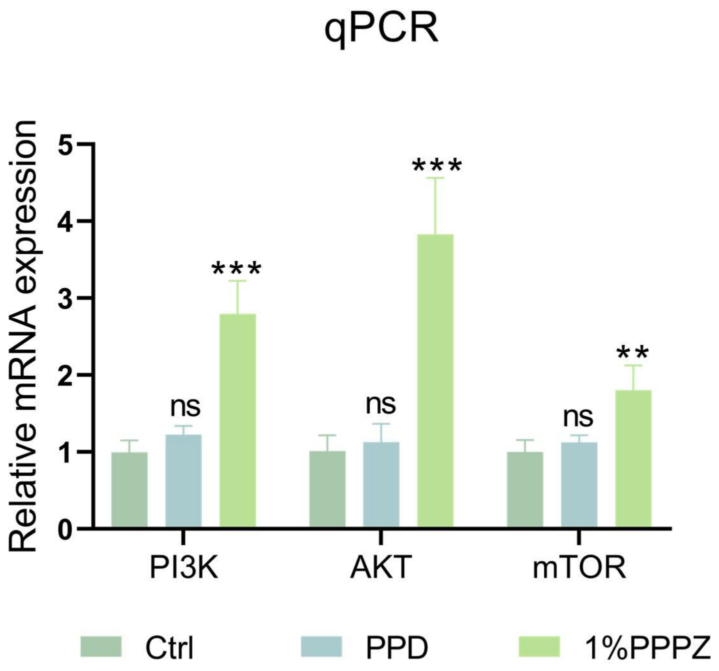

Fig. S25 Detection of relative mRNA expression levels of PI3K/AKT/mTOR pathway genes following various treatments by qPCR.

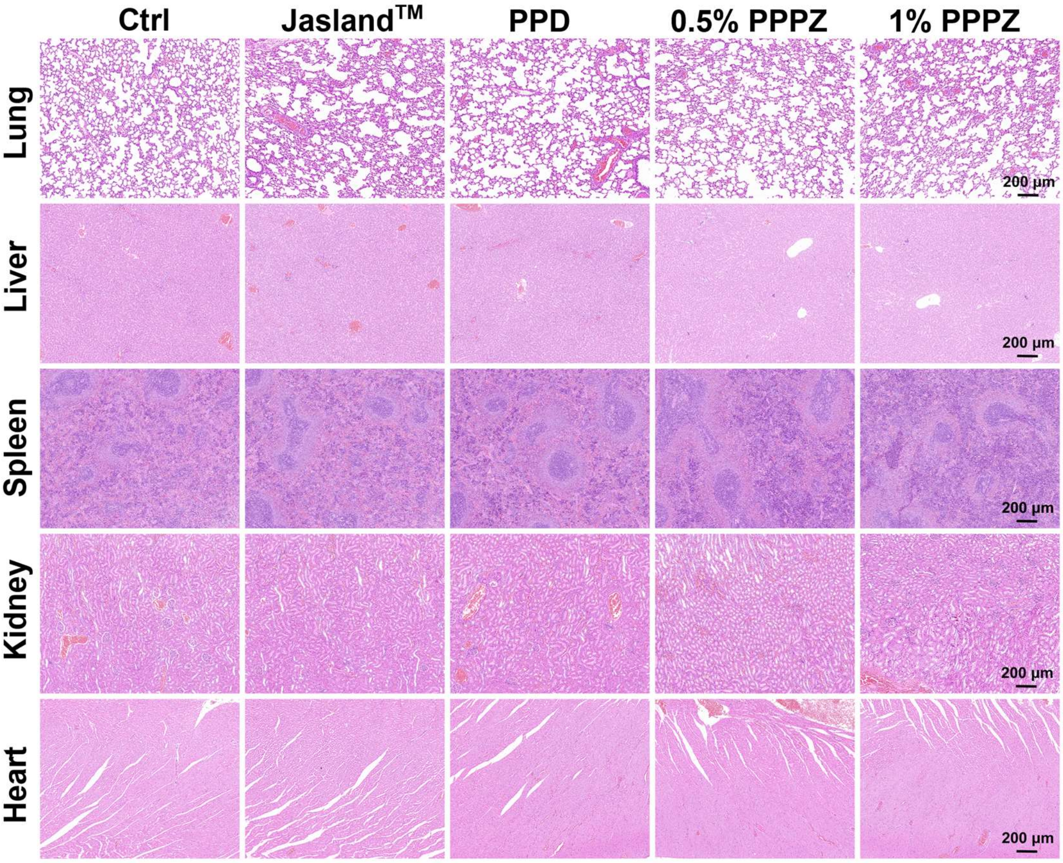

Fig. S26 H&E staining images of major organs after treatment, respectively. Scale bars: 200 μm.

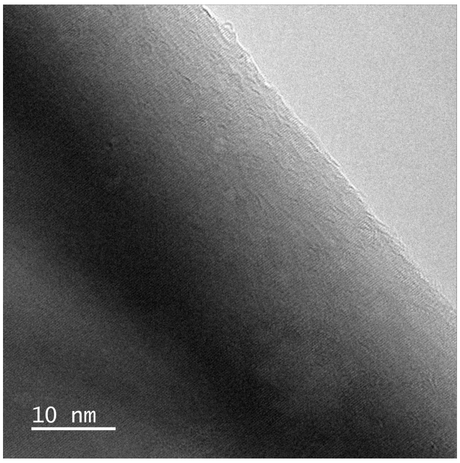

Fig. S27 TEM images of ZnO-O/PDA.

Due to the low O_V concentration, there is no apparent coating layer on the surface of the ZnO-O, indicating that PDA did not form extensively on the surface.

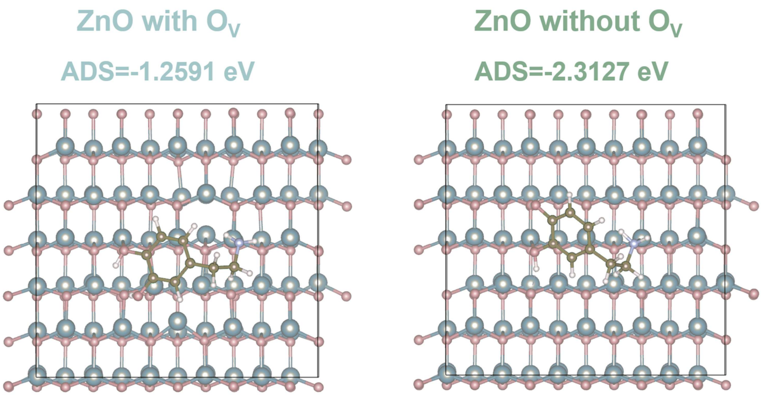

Fig. S28 DA adsorption energy of ZnO with O_V and pure ZnO

The results indicated that in the absence of O_V, the adsorption energy is higher, suggesting that DA monomers are less likely to desorb from the interface without O_V defects. This would prevent the exposure of reactive sites. Conversely, the lower adsorption energy of O_V defects allows water and oxygen to more easily access the reactive sites, maintaining the entire catalytic process in a dynamic equilibrium.

Table S1. Primers sequences for qPCR.

Gene	Sequences
PI3K	GATTCTCAGCAGCCAGCTCTGAT
	GCAGGCTGTCGTTCATTCCAT
AKT	GACAACCGCCATCCAGAC
	CCAGGGACACCTCCATCTC
mTOR	ACTCGCTTCTATGACCAACTGA
	TTTCCATGACAACTGGGTCATTG
GAPDH	CTGCCAACGTGTCAGTGGTG
	TCAGTGTAGCCCAGGATGCC
